# A multi-omics approach to visualize early neuronal differentiation from hESCs in 4D

**DOI:** 10.1016/j.isci.2022.105279

**Published:** 2022-10-04

**Authors:** Athina Samara, Mari Spildrejorde, Ankush Sharma, Martin Falck, Magnus Leithaug, Stefania Modafferi, Pål Marius Bjørnstad, Ganesh Acharya, Kristina Gervin, Robert Lyle, Ragnhild Eskeland

**Affiliations:** 1 Division of Clinical Paediatrics, Department of Women’s and Children’s Health, Karolinska Institutet, Solna, Sweden; 2 Astrid Lindgren Children′s Hospital Karolinska University Hospital, Stockholm, Sweden; 3 PharmaTox Strategic Research Initiative, Faculty of Mathematics and Natural Sciences, University of Oslo, Oslo, Norway; 4 Department of Medical Genetics, Oslo University Hospital and University of Oslo, Oslo, Norway; 5 Institute of Clinical Medicine, Faculty of Medicine, University of Oslo, Oslo, Norway; 6 Department of Informatics, University of Oslo, Oslo, Norway; 7 Department of Molecular Medicine, Institute of Basic Medical Sciences, Faculty of Medicine, University of Oslo, Oslo, Norway; 8 Department of Biosciences, University of Oslo, Oslo, Norway; 9 Division of Obstetrics and Gynecology, Department of Clinical Science, Intervention and Technology (CLINTEC), Karolinska Institutet, Alfred Nobels Allé 8, SE-14152 Stockholm, Sweden; 10 Center for Fetal Medicine, Karolinska University Hospital Huddinge, SE-14186 Stockholm, Sweden; 11 Pharmacoepidemiology and Drug Safety Research Group, Department of Pharmacy, School of Pharmacy, University of Oslo, Oslo, Norway; 12 Division of Clinical Neuroscience, Department of Research and Innovation, Oslo University Hospital, Oslo, Norway; 13 Centre for Fertility and Health, Norwegian Institute of Public Health, Oslo, Norway

**Keywords:** Neuroscience, Cell biology, Omics

## Abstract

Neuronal differentiation of pluripotent stem cells is an established method to study physiology, disease, and medication safety. However, the sequence of events in human neuronal differentiation and the ability of *in vitro* models to recapitulate early brain development are poorly understood. We developed a protocol optimized for the study of early human brain development and neuropharmacological applications. We comprehensively characterized gene expression and epigenetic profiles at four timepoints, because the cells differentiate from embryonic stem cells towards a heterogeneous population of progenitors, immature and mature neurons bearing telencephalic signatures. A multi-omics roadmap of neuronal differentiation, combined with searchable interactive gene analysis tools, allows for extensive exploration of early neuronal development and the effect of medications.

## Highlights

• Multi-omics charting a new neuronal differentiation protocol for human ES cells
• Single-cell analyses reveal marker genes during neuronal differentiation
• Identified transcriptional waves similar to early human brain development
• Searchable tools to visualize single-cell gene expression and chromatin state

## Graphical abstract

**Figure:**
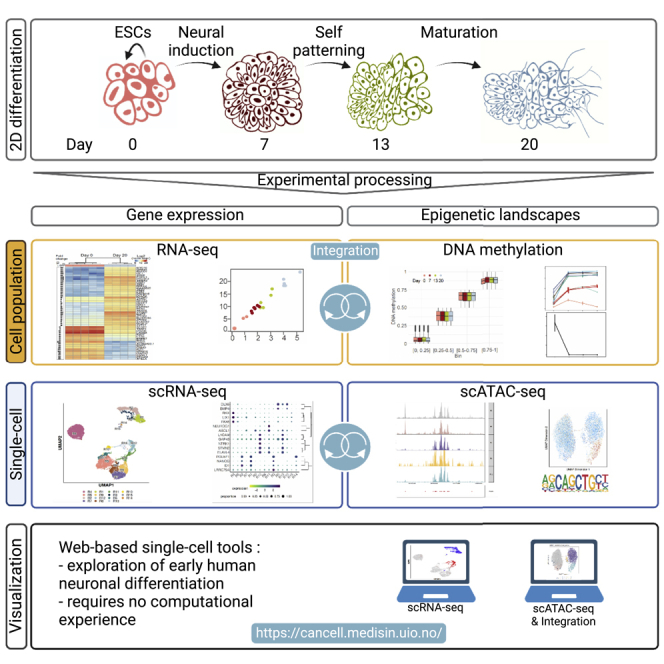

## Introduction

Brain region and cell-specific transcriptional and epigenetic landscapes are fundamental for investigating disease mechanisms and therapeutic interventions. However, the role of epigenetic regulation on the establishment and maintenance of cellular identity during early neuronal differentiation is not well understood (Sun et al., 2021; Yao et al., 2016). Studies of chromatin modifications and global open chromatin in early human brain, neuronal differentiation of human embryonic stem cells (hESCs) and organoid model systems have been insightful on the regulation of neuronal gene programs (Luo et al., 2016; Markenscoff-Papadimitriou et al., 2020; Reilly et al., 2015; Xie et al., 2013). Single-cell resolution analyses have further subtyped the neuronal cells present in human fetal brain development and organoids (Amiri et al., 2018; Eze et al., 2021; Trevino et al., 2020, 2021; Ziffra et al., 2021).

Developmental trajectories can only be spatiotemporally resolved by single-cell omics systematic studies which characterize cells at intermediate differentiation timepoints. Although neuronal differentiation of pluripotent stem cells (PSCs) is an established method to study early development, disease and neurotoxicity (Riemens et al., 2018), fewer omics studies have targeted 2D neuronal differentiation from hESCs and how well they recapitulate early human brain development.

Applying dual SMAD/WNT signaling inhibition for neural induction of hESCs (Cakir et al., 2019; Chavali et al., 2020; Major et al., 2016; Ohashi et al., 2018; Tchieu et al., 2017) we developed a 2D differentiation protocol towards a heterogeneous population of progenitors and neurons bearing telencephalic signatures (Samara et al., 2022).

Combining RNA-seq, global DNA methylation, single-cell RNA-seq and ATAC-seq data and analyzing the integration across four timepoints (4D analysis), we constructed a molecular timeline and correlated transcription factors (TFs) with time- and population-specific chromatin states in hESCs, early fate commitment and during differentiation. We provide access to the single-cell data in user-friendly, interactive web applications that enable visualization of gene cluster regulation during the neuronal differentiation protocol for novel insights and as a basis for future studies. This integrative analysis delineated transcription programs and identified over 26,000 putative cis regulatory elements that link to expressed genes during 2D neuronal differentiation.

## Results

### Initial validation of the neuronal differentiation protocol

**Figure 1:**
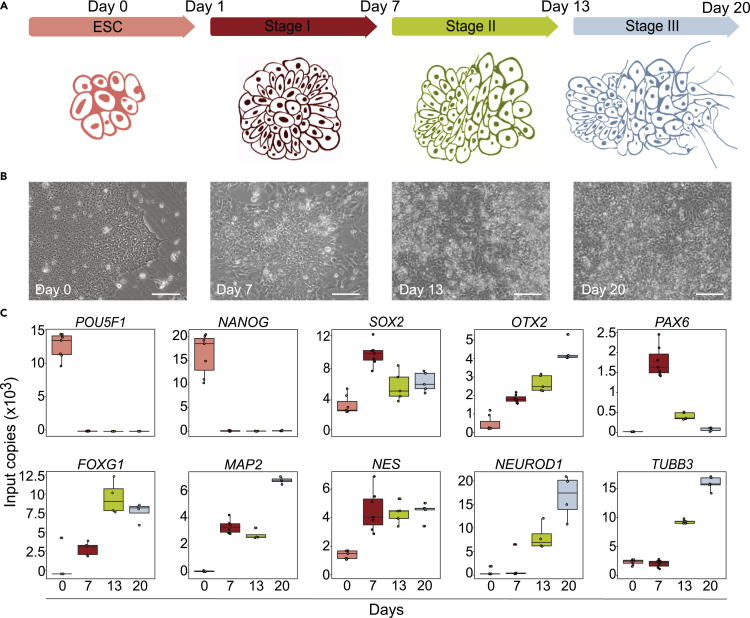
2D protocol with neural induction followed by self-patterning and maturation (A) Schematic illustration of the 20-day timeline of the neuronal differentiation protocol from hESCs. (B) Representative 20x brightfield phase contrast images of hESCs at Days 0, 7, 13 and 20 (scale bar 100 μm). (C) ddPCR results from 4-6 replicates of mRNA expression of selected marker genes from Days 0, 7, 13 and 20. See also Figure S1.

After establishing the 2D neuronal differentiation protocol for hESCs (Samara et al., 2022) we analyzed the hESCs (Day 0) and derivative cell populations at three timepoints. We empirically defined the end of the neural induction phase (Stage I) at Day 7, the end of the self-patterning phase (Stage II) at Day 13 and the end of the maturation phase (Stage III) at Day 20 (Figure 1A). For the neural induction of unsynchronized HS360 hESCs (Main et al., 2020; Ström et al., 2010), the LSX cocktail (LDN193189, SB431542 and XAV939), antagonizes BMP, TGFβ and WNT signaling pathways driving cells to anterior neuroectoderm (Cakir et al., 2019; Major et al., 2016; Ohashi et al., 2018; Tchieu et al., 2017). By the end of Stage I, cells form thickened neural rosettes, and self-pattern at Stage II before the final Stage III FGF2/EGF-induced maturation (Figure 1B). Despite absence of inhibitors at the self-patterning stage II, the anterior forebrain identity is retained, and cells proceed to maturation, as shown by the ddPCR results (Figure 1C).

Neural induction significantly decreased expression of the pluripotency transcription factors (TFs) *POU5F1* and *NANOG* (p < 0.00001). At Day 7 expression of the early neural markers *SOX2* and *NES* increased and stabilized, whereas *PAX6* expression peaked at Day 7 before decreasing significantly at Days 13 and 20 (p < 0.0001). Expression of *OTX2*, the TF which regulates neurogenesis and antagonizes ground state pluripotency, the late onset pan-neuronal marker *TUBB3* and also *MAP2 and FOXG1*, increased as cells differentiated. Immunofluorescence imaging of OCT4, OTX2, SOX2, PAX6, NESTIN and TUBB3 showed localization with protein expression levels correlating with ddPCR (Figure S1).

### Global expression profiles reveal neuronal differentiation and maturation signatures

**Figure 2:**
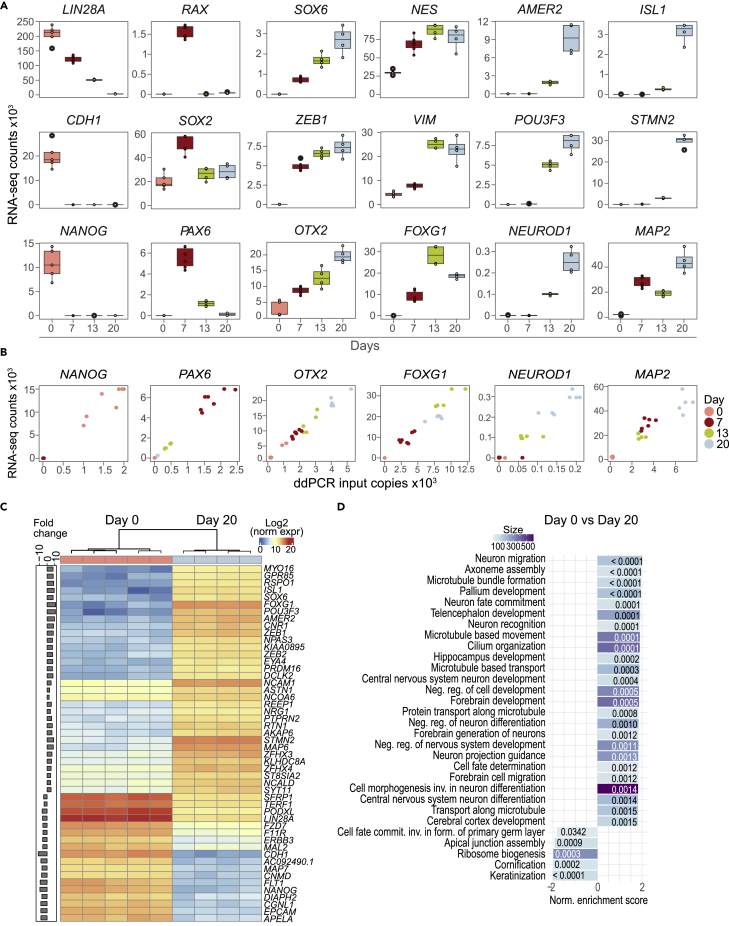
Global RNA-seq and correlation to ddPCR and GO analyses results (A) Normalized gene expression counts for selected genes showing transcriptome expression patterns from loss of pluripotency towards neuronal maturation. (B) Scatter plots of RNA-seq and ddPCR for marker genes *NANOG*, *PAX6*, *OTX2*, *FOXG1*, *NEUROD1* and *MAP2* at Days 0, 7, 13 and 20. (C) Heatmap of top 50 differentially expressed genes between Days 0 and 20 replicates. Fold change is shown to the left. (D) GSEA analysis of differentially expressed genes from Days 0 and 20. See also Figure S2 and Tables S1 and S2.

To increase gene expression sensitivity, we performed bulk gene expression analysis with higher sequencing depth (Figures 2 and S1). Overall, we found 11,313 differentially expressed genes (DEGs) comparing cells from Day 0 to 20 (Table S1). More genes were differentially expressed during neural induction (Day 0 to 7), compared to the later stages, with self-patterning (Day 7 to 13) and maturation (Day 13 to 20) stages (Table S1). The most extensive transcriptional changes occurred between Day 0 and Stage I, with loss of pluripotency and gain of neuralization markers (Figures 2A, S2A, and S2F). We confirmed that bulk RNA-seq analysis for selected marker genes correlates well with ddPCR (Figures 2B and S2G).

Specific gene expression patterns drive the loss of pluripotency towards neuronal maturation (Figure 2). These may be steep decreases after neuronal induction, as seen for *LIN28A* and *CDH1*, expression peaking at Day 7 or Day 13 (such as *RAX* and *FOXG1*, respectively) or gradual increase for genes, such as *OTX2* and *SOX6* (Figures 2A, S2A, and S2B). Moreover, the expression of neuroectodermal patterning Wnt/β-Catenin negative regulator *AMER2* (Pfister et al., 2012), and neuronal differentiation marker *STMN2* (Wang et al., 2019b) increase at Day 13, and increase further at Day 20 (Figures 2A, S2D, and S2E). On Day 20 we also find genes correlated to specific neuronal types, such as *GRIA1*, and *GNRH1* (Figure S2F) and the top 50 differentially expressed genes between Days 0 and 20 were plotted as a heatmap (Figure 2C).

We further identified biological processes (BP) enriched among the DEGs with gene ontology (GO) analyses (Figure 2D; Table S2), which revealed upregulated BPs related to neuronal maturation from Day 0 to 20 (Figure 2D). Stage-specific GO analyses revealed enrichment of BPs involved in neurogenesis and neuron development differentiation at stage I, and BPs involving synaptic organization and neurotransmitter regulation and secretion at the end of the maturation stage (Table S2).

### DNA methylation correlates with neuronal transcriptional programs during differentiation

**Figure 3:**
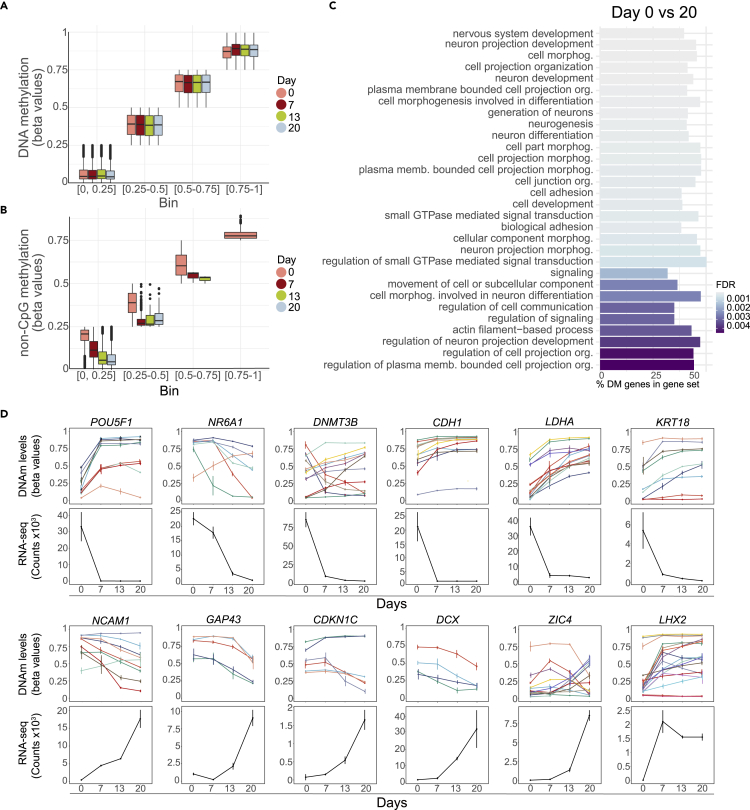
Specific DNAm changes during neuronal differentiation (A) Mean DNAm levels for each sample across all CpGs and non-CpGs (grouped in bins of 0.25) at Days 0, 7, 13, and 20. (B) Mean DNAm levels for each sample across all non-CpGs (grouped in bins of 0.25) from Days 0, 7, 13 and 20. (C) GOMETH analysis of top 30 BPs based on top 10% DMCs for Day 0 to 7. (D) Significant CpGs of gene expression (derived from MORE) for Days 0, 7, 13 and 20. Top panels show DNAm mean +/− standard deviation whereas bottom panels show normalized RNA-seq counts +/− standard deviation for selected genes. See also Figure S3 and Tables S1 and S3.

DNAm in human cells is mainly restricted to CpG sites and is essential for normal development (Smith and Meissner, 2013). As hESCs transition to differentiated neurons, dynamic DNAm changes regulate gene expression and the establishment of cell-type specificity (Stricker and Götz, 2018). To assess DNAm in the present protocol, we identified CpGs which are differentially methylated (DMCs) between Day 0, 7, 13 and 20 (Figures 3 and S3). As expected, comparing Day 0 and 20 reveals massive DNAm changes (n = 210,049 DMCs, Table S1). Although we observe major changes in DNAm during the differentiation protocol (Table S1), the bulk DNAm levels and the distribution of unmethylated and methylated CpGs remains the same across all four timepoints (Figures 3A, S3B, and S3C).

Deconvoluting these changes temporally, the highest number of DMCs was observed between Day 0 and 7 (n = 161,600), with fewer changes in the self-patterning phase (Days 7–13, n = 39,545) and during cell maturation (Days 13–20, n = 47,676) (Table S1). Next, we used GOMETH analysis (Maksimovic et al., 2021) to explore shared biological functions among DMCs. In line with the gene expression results, from Day 0 to 20 we observed enrichment of BPs involved in neurogenesis, brain development and others (Figure 3C) showing how DNAm modulates neuronal differentiation (Figure 3A). Similarly, these analyses identified DMCs between Day 0 to 7 and Day 0 to 13, with BPs involved in neuron projection morphology, which fits well with the cell transitions (Figures S3D and S3E). One of the most significant GO terms is “neuron migration”, evidenced by expression of genes such as *DCX* and its partner *PAFAH1B* (Nadarajah and Parnavelas, 2002) (Figure 3).

To explore the correlation between DNAm and gene expression, we combined the DNAm and RNA-seq data sets based on CpG probe location and gene locus (Figures 3D, S3F, and S3G; Table S1). Of the Stage I gene annotated DMCs, 72% overlap with differentially expressed genes, inferring functional impact on gene expression. For genes with DMCs we generally observed a decrease on transcriptional activation or an increase for genes becoming repressed during the course of differentiation. The expression levels of the majority of the differentially expressed genes between Day 0 and 20 are predicted to be associated with DNAm changes (8,011 of the 11,313 DEGs). The expression of markers of late trophectoderm (e.g., *KRT18*), pluripotency maintenance (*POU5F1*), suppression of pluripotency (*NR6A1*) (Wang et al., 2016), metabolic reprogramming (*LDHA*) (Zheng et al., 2016), or spatiotemporally regulated cortical TFs and cell cycle related genes (*LHX2*, *CDKN1C*) (Chou and Tole, 2019; Laukoter et al., 2020) and neuronal differentiation and maturation markers, (such as *DCX*), may be regulated by one or more CpGs (Figures 3C, S3F, and S3G; Table S3).

Methylated non-CpGs (mCpHs) have been associated with transcriptional repression in the mouse genome (Xie et al., 2012), and also detected in human ESCs, brain and organoid models (Lister et al., 2009; Luo et al., 2016; Schultz et al., 2015). We demonstrate that the distribution of methylated non-CpGs vary across timepoints (Figure 3B). Non-CpG DNAm levels were enhanced at Day 0 cells and declined during differentiation (Figure 3B), similar to the decline observed during differentiation of hESC to cerebral organoids (Luo et al., 2016).

### Identification of heterogeneous populations of progenitors, mature and immature neurons with telencephalic signatures

**Figure 4:**
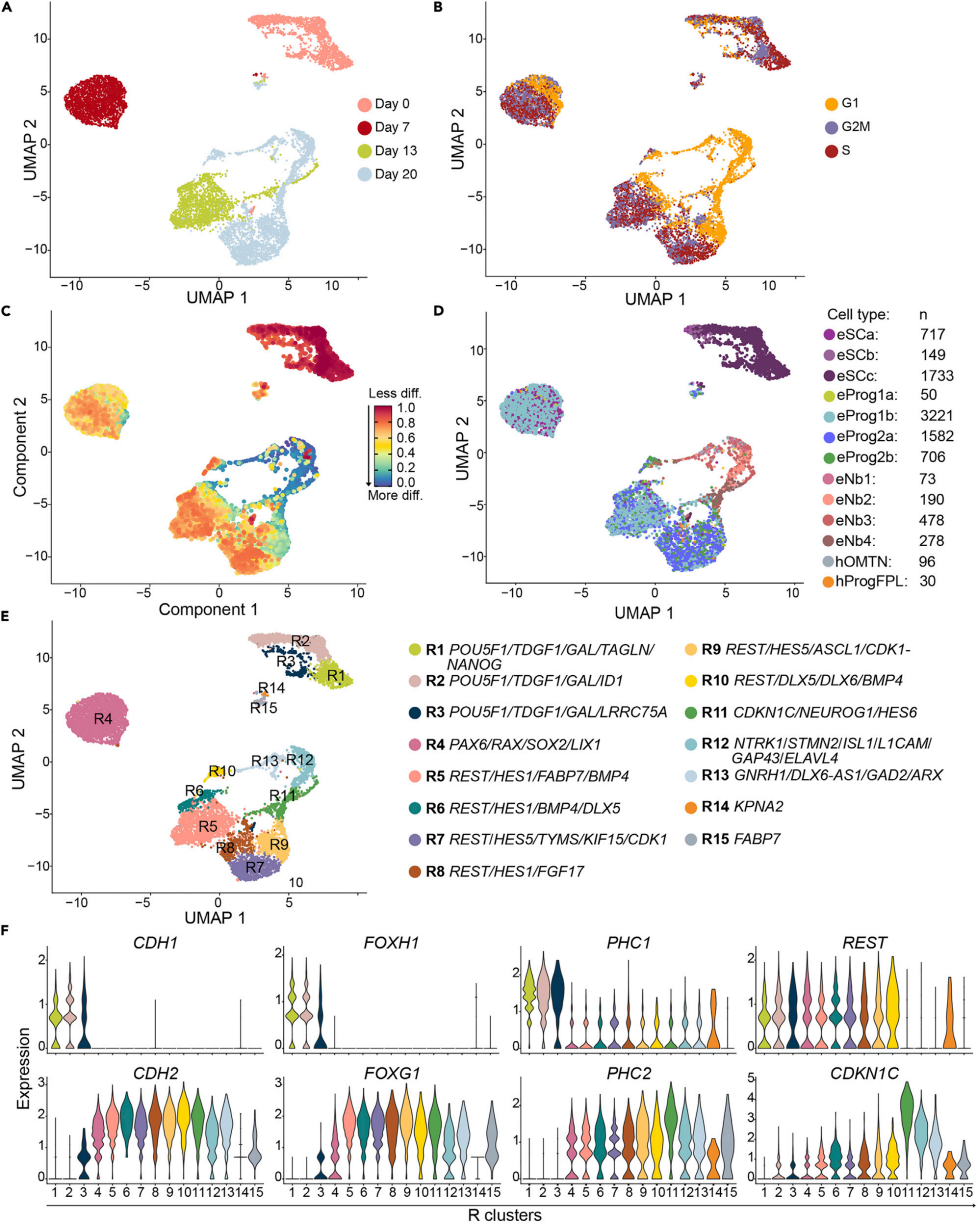
Identification of cell populations during neuronal differentiation of hESCs (A) UMAP representing single-cell RNA-seq clusters per timepoint. (B) UMAP of cell cycle analysis showing all cells analyzed and colored by assigned cell cycle phase. (C) The inferred neuronal differentiation trajectory using CytoTRACE, where less differentiated cells are shown in red and more differentiated cells are shown in blue. (D) A UMAP projection based on SingleR cell annotation to the La Manno Brain dataset. The La Manno Brain dataset is comprised of merged data derived from human embryo and *in vitro* differentiated hESCs. Cell types starting with “e” are hESC derived cells, whereas cell types starting with “h” are *in vivo* human embryo cell types. Nb1-4; neuroblast, Prog1-2; progenitor, SCa-c; stem cells, Gaba; GABAergic neurons, NbGaba; GABAergic neuroblasts, NbM; medial neuroblasts, OMTN; oculomotor and trochlear nucleus, ProgFPL; progenitor lateral floorplate. (E) The UMAP representing cell clusters R1 to R15 with corresponding gene annotations mapped at resolution 0.55. The clusters are indicated by different colors and gene annotations per cluster are given after each color corresponding bullet below the UMAP. (F) Violin plots representing gene expression levels and distribution in clusters R1 to R15 for selected genes. See also Figure S4 and Table S4.

To characterize the gene expression signatures, composition, differentiation pathway trajectories and the maturation level of the cell types derived, we performed single-cell RNA-seq (scRNA-seq) analyses at Days 0, 7, 13 and 20 (Figures 4 and S4; Table S4). We used the batch correction algorithm Harmony on the unfiltered data and found our data to be very consistent across replicates (Figures S4D and S4E). Moreover, differential expression analyses between days were performed to investigate if DEGs were comparable between bulk RNA-seq and scRNA-seq (Table S4). We found an overlap of genes between DEGs for global RNA-seq and scRNA-seq of 62–91%, confirming a high correlation between the two datasets. The scRNA-seq data can be visualized in the open access webtool “hESC Neuronal Differentiation scRNA-seq” (hESCNeuroDiffscRNA) where expression of genes can be explored per cell, cluster and timepoint. A total of 9,337 cells from two time-course experiments were aggregated and projected in UMAPs, 1,900 Day 0 cells, 2,368 Day 7cells, 2,045 Day 13 cells and 3,024 Day 20 cells) (Figures 4A and S4 and hESCNeuroDiffscRNA, cell information tab, orig.ident).

### Inferring quantitative analysis of cell cycle phase

Neuronal development involves major cell cycle alterations. G1-phase lengthening is associated with the transition to more differentiated cell types, whereas S-phase duration is linked to progenitor cell expansion (Arai et al., 2011). The cell cycle-specific gene trajectories documented the decreased proportion of cells in S phase and an increased proportion of cells in G1 phase as cells transition from Day 0 to 20 (Figures 4B and hESCNeuroDiffscRNA). Although, terminally differentiated neurons are found in G0 (not in G1), and the analyses tools currently available cannot accurately predict cells in the G0 phase, the results are consistent with previous studies showing that maintenance of proliferation and pluripotency in PSCs, neural stem cells and progenitor cells are cell cycle-regulated (Becker et al., 2006; Boward et al., 2016; Liu et al., 2019; Soufi and Dalton, 2016). The cell cycle regulator *CDK1* was expressed by 60% Day 0 cells, 45% at Day 7, 53% at Day 13, and reduced to 33% at Day 20 (Figure S4G, % from the hESCNeuroDiffscRNA). Moreover, utilizing CytoTRACE to predict single-cell hierarchies (Gulati et al., 2020) showed that cell potency gradually decreased from Day 0 to 20 (Figure 4C), confirming the cell cycle phase prediction.

### Development and differentiation markers used for cluster resolution and annotation

For a preliminary assessment of the cell types in our neuronal differentiation dataset, we utilized a scRNA-seq Human Brain dataset (La Manno et al., 2016) and RNA-seq data from Human Primary Cell Atlas (Aran et al., 2019) (Figures 4D and S4F). According to the Human Primary Cell Atlas, D0 hESCs overlap with ESCs and IPSCs, whereas the majority of D7, D13 and D20 cells overlap with neuroepithelial cells (Figure S4F). When we compared our datasets to the La Manno Brain Data, the D0 cell population correlated with ESCs, and D7, D13 and D20 cells that are dividing and REST positive (Figures 4B and 4F) annotate to human neuronal progenitors whereas the cells in the D20 clusters R11-13 correlated with neuroblasts (Figure 4D).

The four timepoints were resolved into 15 clusters (R1-R15, Figures 4E and S4B). Corresponding cell numbers per cluster and cells per timepoint per cluster are shown (Table S4). Based on expression, the genes *POU5F1*, *TDGF1*, *GAL*, *LRRC75A*, *RAX*, *LIX1*, *TYMS*, *HES1*, *HES5*, *HES6*, *FGF17*, *DLX5*, *DLX6*, *GAP43*, *STMN2* and *GNRH1* were used for R1-R13 cluster annotation (Figure 4E). For R14, consisting of 27 Day 0 cells, we used *KPNA2*, a gene associated with OCT4 localization (Li et al., 2008). For R15, a pool of 90 cells from Days 7, 13 and 20, we used *FABP7*, which is expressed in NSCs during development (Kurtz et al., 1994).

### Characterizing the unsynchronized hESC population

We identified three Day 0 clusters (R1-3) where all cells expressed *POU5F1* (Figure S4G) verifying their pluripotency. TFs essential in establishing and maintaining pluripotency (i.e., *GAL*, *TDGF1*, *ID1*, *FOXH1* and *SOX2)* were highly expressed in clusters R1-3 (Figures S4F and S4G, as can be observed hESCNeuroDiffscRNA). R3 cells expressed the highest levels of *LRRC75A* (Figure S4G). As others have reported (Chen et al., 2021, p. 1), *PHC1* was highly expressed in hESC clusters R1-R3, and its expression was greatly reduced in differentiating cells. Downregulation of *PHC1* was compensated by increased *PHC2* expression, indicating a role for PHC2 in human neuronal differentiation (Figures 4F and S7B). Focusing on the *FOX* family of TFs, a clear switch was observed from *FOXH1* expression in R1-R3, to the expression of the master regulator of brain development *FOXG1* (Beyer et al., 2013; Chiu et al., 2014) in all other clusters (Figures 4F and S5B).

## LSX forebrain induction cues evident at the end of stage I

Under LSX induction, hESCs undergo morphogenetic events and form neural rosettes. These Day 7 cells mapped to a single cluster (R4, Figure 4F), enriched in the rostral marker *LIX1* (Figure S4G). In contrast, expression of the preplacodal genes *EYA1* and *SIX1* (Ikeda et al., 2007; Schlosser, 2014) *and* of the caudal markers *PAX5* and *GBX2* (Kirkeby et al., 2012; Maroof et al., 2013) was low throughout differentiation, confirming that the LSX efficacy is enabling cell-fate commitment persistence.

Upon neural induction, the key neural TF PAX6 is upregulated and interacts with SOX2 (Zhang et al., 2019, p. 2). R4 cells were *SOX2* positive showing high *PAX6* and *RAX* expression (Figure S4G), and express neuronal rosette markers, such as *DACH1*, *POU3F2*,*NR2F1* and *NR2F2* (Fedorova et al., 2019). A distinct switch from *CDH1* (Epithelial Cadherin) to *CDH2* (Neural Cadherin) expression was observed (Figure 4F), and other developing forebrain specification and differentiation stage markers (such as *OTX2*, *HESX1*, *FOXG1*, *LIN28A*, and *FABP7*) were detected.

### Self-patterning does not affect fate commitment

At Day 13, which marks the end of the self-patterning stage, 75% of the cells mapped to cluster R5 and most of them expressed *REST* (Figures 4F and S4G). *SIX3*, *DLX5* and *BMP4* (Figure S4G) were expressed in the *FGF8*/*HES1* enriched R6 cells. Moreover, R6 was enriched in *TAGLN*, but absent in the R5 cells co-expressing *NKX2*.*1* and *SOX6*. *CNTN1*, a potent inducer of neuronal migration and Notch ligand (Hu et al., 2003) and potent inducer of neuronal migration (Lee et al., 2014), was exclusively expressed in R6 cells negative for *NTN1*, *DLL1*, *FABP7* and *POU3F2*. Comparing to results of 8 week human embryonic tissue (Kirkeby et al., 2012), no midbrain and hindbrain markers were detected at Day 13, confirming that the self-patterning phase does not affect fate commitment.

### Characterization of the day 20 heterogeneous population

Day 20 cells retained their identity and clustered in R7-13 (Figure S4G). Some cells expressed high levels of *CDK1* (Figure S4G), whereas other cells were still regulated by *REST* and expressed *DLX5* and *CDKN1C*. *CDKN1C*, which forms complexes with histone deacetylases to repress neuronal genes in non-neuronal cells (Laukoter et al., 2020) is inversely correlated with *REST* expression and enhanced in R11-13 (Figure 4F). Of interest, *ARX*, a regulator of cortical progenitor expansion by repression of *CDKN1C* (Colasante et al., 2015) was only expressed in R13 cells (Figure S4G). Neuronal differentiation correlated with *CDK6* upregulation and G1 shortening. *CDK6* is directly regulated by *GLI3* and expression of *GLI3* (Hasenpusch-Theil et al., 2018) (detectable at R4) dropped significantly in R12-13. *REST* is known to be downregulated during neurogenesis and in differentiating neurons and the pattern was recapitulated in this study (Figure 4F).

### Neuronal maturation signatures

Day 20 cells were highly enriched for *MAP2*, and clusters R11-13 were enriched for *DCX*, which is a marker of migratory neurons. Genes expressed in proliferating neuroblasts associated with cortical migration control and developing rostral brain structural patterning, such as *EMX2* (Pang et al., 2008; Spalice et al., 2009; Verrotti et al., 2010), decreased in clusters R11-R12 and were undetectable in R13 (Figure S4G). FGF8, an anterior-posterior patterning molecule, acting mainly via EMX2 repression (Hao et al., 2019, p. 8), was expressed in R4 and R6 cells and in a few Day 20 cells, mainly in R8 and R10 clusters. Furthermore, *FGF17* (Figure S4G) and *FGF18* were mostly expressed at Day 20 R8 cluster. *HES6*-enriched cluster R11 (Figure S4G) was composed of Day 13 and 20 cells, and most of the R11 *NEUROG1-*negative cells were Day 13 cells. Neural stem and progenitor marker *ZEB1*, which was downregulated on neuronal differentiation to permit proper migration of immature neurons (Wang et al., 2019a), was expressed in almost all cells (Figure S4G). In addition, *FOXG1*-enriched R13 cells also express high levels of *DLX5* (Figure S4G).

Of note, the expression of *GNRH1* (Gonadotropin Releasing Hormone 1) was expressed in 30% of the R12-13 cells (9% of Day 20 cells) (Figure S4G). Of these cells, some expressed GABAergic or glutaminergic processing enzymes. As the mechanisms that contribute to the development of extrahypothalamic GnRH neurons are not fully described, such data are vital for studies of development, puberty and reproduction.

### Chromatin accessibility analysis identifies regulation signatures during differentiation

**Figure 5:**
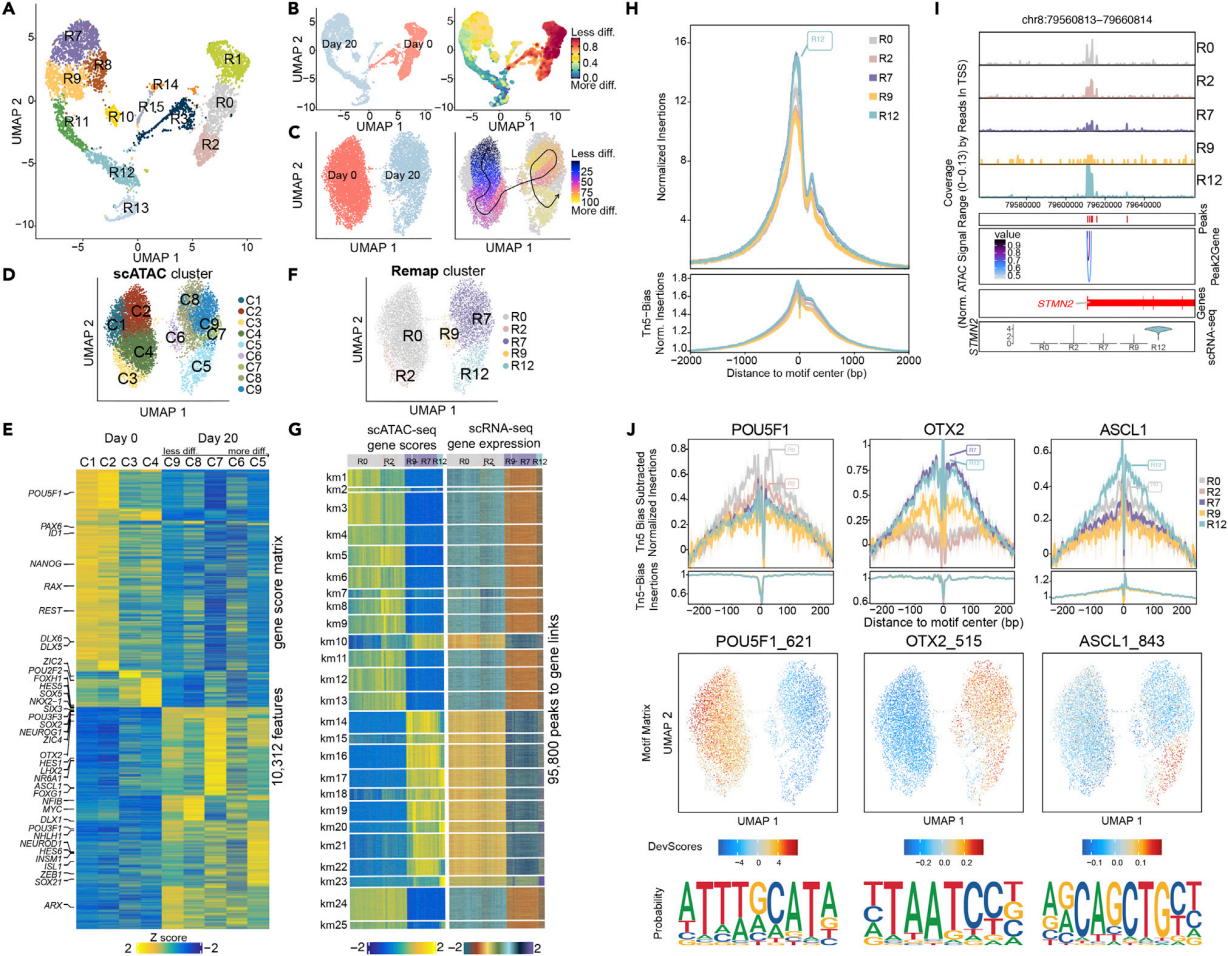
Integration of single-cell chromatin opening with scRNA-seq during neuronal differentiation (A) Multidimensional reduction UMAP plot of scRNA-seq corresponding to the timepoints used for scATAC-seq analysis. (B) UMAP plot showing original identity of cells at scRNA-seq modality and corresponding differentiation trajectory. (C) UMAP plot showing original identity of cells at scATAC-seq modality and corresponding supervised pseudotime trajectories. Visualization of the pseudotime trajectory by an arrow from ESC cluster C1, C2 via C3, C4, and to Day 20 clusters C6, C7, C9, C8 before it crosses over and finally ends in C5. Sale bar less differentiated to more differentiated. (D) UMAP plot showing clusters at scATAC-seq modality (C1-C9). (E) Top selected marker genes from scRNA-seq data shown on a heatmap plot computed on Gene Score Matrix. Days and scATAC-seq clusters are indicated on the top. (F) Remap UMAP plot of renamed clusters following constrained alignment of cell populations after integration of scATAC-seq and scRNA-seq. (G) Peak to gene linkage heatmap for gene scores scATAC-seq and corresponding scRNA-seq gene expression are represented side-by-side. Columns are annotated with colors above for integrated clusters R0, R2, R7, R9 and R12. Rows were clustered using k-means clustering (k = 25). A regulatory region can be linked to multiple genes, and a gene can be linked to many regulatory regions. Z-scores (−2 to 2) are shown below each heatmap. (H) Chromatin openness of integrative cluster R0, R2, R7, R9 and R12 overall TSS. (I) Tracks shown on peak browser for selected gene *STMN2* on integrated cell clusters. Bottom panel shows co-accessibility interactions around TSS. (J) Motif footprinting for selected TFs POU5F1, ASCL1 and OTX2 demonstrating preferential opening in different cell clusters. The middle panel shows the corresponding motif deviation scores of ArchR identified TFs POU5F1, ASCL1 and OTX2. The scores are calculated for each TF motif observed in an accessible region and in each cell for the deviation from expected average accessibility across all the cells. The representative sequence logos identified in accessible regions across the dataset are shown below. See also Figure S5.

In line with the bulk RNA-seq data, the scRNA-seq results showed downregulation of pluripotency genes and upregulation of brain development genes correlating with neuronal transcriptional programs during differentiation. To further assess the epigenetic landscape changes on differentiation, we performed single-cell assay for transposase accessible chromatin sequencing (scATAC-seq). The analysis focused on chromatin-based gene regulation from loss of pluripotency at Day 0 to Day 20 (Figure 5 and S5).

### Reanalyzing scRNA-seq datasets for integration with scATAC-seq data

To integrate scATAC-seq and scRNA-seq, the scRNA-seq datasets for Day 0 and Day 20 were reanalyzed. 1910 Day 0, and 3033 Day 20 cells were projected in 13 clusters (Figure 5A) and in accordance with the maturation trajectory seen in the corresponding CytoTRACE plot (Figure 5B). scRNA-seq clusters were numbered and annotated, cohering to the initial four-timepoint analysis (Figures 5A, S5A, and S5B). Thus, Day 0 cells resolved into five clusters (R0-3 and R14) whereas Day 20 clusters were resolved into nine clusters (R7-13 and R15).

### Chromatin accessibility changes globally during differentiation

The analysis of 4,901 Day 0 nuclei and 2,847 Day 20 nuclei and the scATAC-seq data showed good distribution of fragment sizes, fragment numbers, and transcriptional start site (TSS) enrichment (Figures S5C–S5F). The supervised pseudotime trajectory scATAC-seq analysis, which predicts paths for gene regulatory changes in cells during differentiation, showed a similar profile to the single-cell gene expression CytoTRACE analysis (Figures 5B and 5C). We mapped four chromatin accessibility clusters at Day 0 (C1-4) and five at Day 20 (C9-5; Figure 5D). Genome tracks demonstrate differential chromatin opening in scATAC-seq clusters for gene loci *POU5F1*, *REST*, *GAD2* and *DCT* (Figure S5G). We next generated a gene score matrix heatmap representing a score of chromatin opening of 200 kb genomic regions. Higher gene scores for genes related to regulation of pluripotency, such as *POU5F1*, *NANOG*, *ID1*, and known enhancer specific binding factors in development, such as *ZIC2* (Hong et al., 2011) were found in Day 0 clusters (C1-C4) (Figures 5E and S5B). *SOX2* is regulated by several enhancers and interacts with multiple but distinct groups of TFs, including POU3 class partners (Iida et al., 2020; Mistri et al., 2015; Tang et al., 2015; Zhu et al., 2014). The scATAC-seq data showed that chromatin accessibility for *SOX2*, *POU3F1*/*BRN1* and *POU3F3*/*BRN3* increased with differentiation clusters (C9-C5) (Figure 5E). Moreover, neuronal genes *SOX21*, *NFIB* (Piper et al., 2014), and the *OTX2* locus which is associated with early neuronal development regulation, showed a more open chromatin organization in neuronal clusters C5-C9. The gene score of *NEUROD1* showed increased chromatin accessibility in neuronal clusters C7-C5 (Figure 5E).

### Correlation of chromatin regulatory dynamics and gene expression

To better connect the regulatory open chromatin with gene expression we performed integrated analysis of scATAC-seq with scRNA-seq using ArchR (Granja et al., 2021). Following constrained alignment of cell populations after integration of scATAC-seq and scRNA-seq, the integrated clusters were renamed to correspond to the previously annotated scRNA-seq clusters (Figures 5F and S5B). Pluripotency clusters C1-C4 remapped to two of the four Day 0 scRNA-seq clusters, R0 and R2 (Figures 5D–5G and S5I). Day 20 clusters C9-C7 mapped to cluster R7, correlating chromatin openness and gene expression in single cells for markers such as *HES1* and *CDK1* (Figures 5D–5G, S5B, and S5I). Cluster C6 mapped to R9, which was marked by expression of REST, HES1 and ASCL1 (Figures 5D–5G, S5B, S5G, and S5I). Endpoint cluster C5 remapped to R12, which has high *STMN2* and *NTRK1* expression Figures 5D–5G, 5I, S5B, and S5I). We assessed scATAC-seq peaks across *TGDF1*, *CDH1*, *CDH2*, *STMN2*, and *DCX* loci across the integrated clusters (R0, R2, R7, R9 and R12) and found cluster-specific chromatin opening correlating with detected level of gene expression in single-cell transcriptome data (Figures 5I and S5J). Furthermore, the peak-to-gene co-accessibility arcs show that the expressed genes *STMN2*, *TDGF1*, *CDH1*, *CDH2* and *DCX* are linked to potential cis regulatory regions (CREs).

To explore the interaction of putative CREs with gene expression, we mapped 95,800 peak-to-gene links grouped into 25 clusters and observed a clear correlation of the predicted CREs and gene expression (Figure 5G). Chromatin accessibility peak annotation analysis revealed varying enrichment across integrated clusters at promoters, intronic, exonic and distal regions (Figure S5H) but with a clear enrichment at TSS in every cluster (Figure 5H). These results agree with previous studies showing that neuronal gene activation in early human brain development depends on multiple distal regulatory regions (de la Torre-Ubieta et al., 2018; Markenscoff-Papadimitriou et al., 2020; Reilly et al., 2015; Trevino et al., 2020, 2021; Ziffra et al., 2021).

To understand the dynamics of lineage-defining factors at pluripotency and differentiation endpoint, we mapped motifs within accessible chromatin regions for TFs found in our analyses (Figures 5J and S5K). Motif footprinting for POU5F1 underlies a regulatory function in accessible chromatin in pluripotent clusters R0 and R2, whereas ASCL1 and OTX2 footprints were more enriched in differentiated clusters R12 and R7. The motifs of POU5F1, DLX6, ASCL1 and OTX2 and motif enrichment in open chromatin in individual cells illustrate how lineage-defining TFs can dynamically regulate gene expression programs during neuronal differentiation (Figures 5J and S5K).

### Molecular signatures of neuronal differentiation

**Figure 6:**
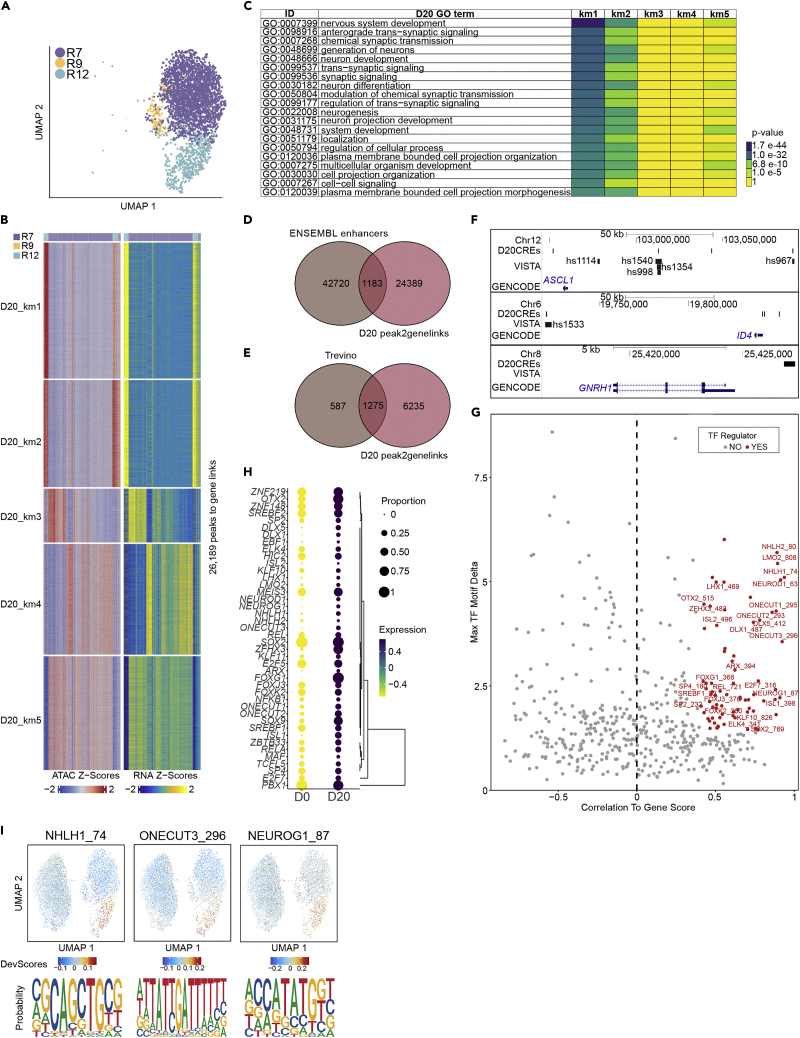
Molecular regulation of neuronal differentiation (A) A constrained scATAC-seq Day 20 UMAP with annotated integrated clusters R7, R9 and R12. (B) Heatmap of chromatin accessibility and gene expression side-by-side representing 26, 189 peak to gene links in Day 20. Columns are annotated with colours above for integrated clusters R7, R9 and R12. Rows were clustered using k-means clustering (k = 5). (C) Gene Ontology analysis by gprofiler representing top 20 significant GO terms of Day 20 linked genes (genes linked with putative CREs having correlation value greater than 0.45 and with significant FDR<1e-4) for k- mean groups 1–5. (D) A venn diagram representing correlation of Day 20 putative CREs with enhancers from Ensembl Human Regulatory Regions (GRCh38.p13). (E) A Venn diagram representing overlap of linked genes from peak-to-gene link analyses of Day 20 versus Trevino linked genes from a single-cell omics study of early human brain development (PCWs 16–24, human cortex). (F) Putative CREs for *ASCL1*, *ID4*, and *GNRH1* are shown together with VISTA enhancers in the UCSC genome browser. (G) A selection of significant positive TF regulators identified on scRNA-seq profiles matched with open chromatin accessible regions computed using gene integration scores with motifs in the putative CREs labeled in red circles. Negative regulators and not significant TFs are labeled with grey circles. X-axis represents negative and positive correlation to gene score and y-axis show max TF delta value. (H) A bubble plot showing expression level in Days 0 and 20 of Day 20 TF regulators. (I) Motif footprinting UMAPs demonstrating preferential opening in different Day 20 cell clusters for NHLH1, ONECUT3, and NEUROG1. See also Figure S6; Tables S5 and S6.

To further elucidate the molecular regulation during neuronal differentiation we focused on Day 20 and linked chromatin accessibility to gene expression to identify potential novel enhancers. We identified 26, 189 putative CREs in five linked groups (k-means clusters 1–5) (Figures 6A and 6B; Table S5). GO enrichment for the linked genes for groups 1–2 and to a lesser degree group 5 were linked to processes such as “nervous system development”, “neurogenesis”, “neuron differentiation” and “anterograde trans-synaptic signaling” (Figure 6C; Table S5). Corresponding GO enrichment for the identified putative CREs in km 1–5 by GREAT (McLean et al., 2010) showed very similar BPs for all groups such as “nervous system development”, “neurogenesis” and “generation of neurons” (Table S5). A total of 1183 potential CREs overlapped with enhancer regions from a collection of Ensembl Human Regulatory Regions (Zerbino et al., 2015; Figure 6D; Table S5). As many of the annotated Ensembl enhancers may operate in other cell types, we next compared our integrated peak-to-gene link Day 20 data with a similar dataset from early human brain. We utilized a single-cell atlas from human cortical development post-conceptional weeks 16–24 and extracted the linked genes from inferred peak-to-gene link pairs after a pseudotime annotation from all five interaction clusters (Trevino et al., 2021) (Figure 6E; Table S5). Of interest, we observed that CRE-linked genes from all the five k-means groups of Day 20 *in vitro* neuronally differentiated cells overlapped with 1275 out of 1862 linked genes from a pseudotime cortical neuron lineage.

Human telencephalon enhancer activity patterns are a subject under intense study (Visel et al., 2013). To address whether any of the putative Day 20 CREs may function as enhancers we compared their genomic location with the coordinates of active VISTA enhancers for *ASCL1* and *ID4*. We show an overlap with enhancers hs967, hs988, hs1354, hs1533 and hs1540 previously described to be active in the brain of transgenic mice, but no overlap was observed for hs1114 (Figure 6F) (Visel et al., 2007, 2013). However, other CREs did not overlap with previously characterized VISTA enhancers and may potentially be new candidate enhancers. For example, we have shown that *GNRH1* was expressed in R12-13 cells and our integrative analysis identified a set of potential CREs 3.5 kilobases upstream of the *GNRH1* locus (Figure 6F). This genomic distance from *GNRH1* is similar to a rat E2 upstream *Gnrh1* enhancer that has been shown together with enhancers E1 and E3 to be important in driving robust neuronal expression (Iyer et al., 2010). To better understand the regulation of neuronal differentiation programs we mapped TF motifs that were enriched in the putative CREs and correlated these with expression in Day 20 cells (Figures 6G and 6H; Table S5). Motif enrichment with higher correlation included neuronal TF regulators such as neuronal marker NHLH1, the ONECUT family known to promote neuronal differentiation (van der Raadt et al., 2019), and proneural factor NEUROG1 with representative sequence logos (Figure 6I; Table S5).

We also generated peak-to-gene links for hESCs and identified 13, 463 putative CREs in five groups (k-means clusters 1–5) (Figures S6A and S6B; Table S6). Because of fewer peak-to-gene links identified in Day 0, the GO functional enrichment analyses of the linked genes in the five groups displayed less overlap and more general processes such as “regulation of cellular process”, “biological regulation” and this was also true for the corresponding GREAT analyses of potential CREs (Figure S6C; Table S6). Furthermore, 388 of the putative CREs overlapped with annotated enhancers from the Ensembl regulome (Figure S6D; Table S6). Reflecting a smaller dataset, fewer TF expressed in hESCs with motifs enriched identified linked putative CREs were identified (Figures S6E and S6F; Table S5). HMGA1, an architectural chromatin protein that previously has been shown to be highly expressed in hESCs and can prevent differentiation (Shah et al., 2012), showed both high expression and motif enrichment in Day 0 cells with representative sequence logo (Figure S6G).

### Exploration of single-cell data using interactive webtools

It is generally necessary to have expertise in bioinformatics for the analysis of large single-cell sequence data analysis. Open-access interfaces based on open-source tools enabled us to make our scRNA- and scATAC-seq data available to more people, abiding by the Findability, Accessibility, Interoperability, and Reusability (FAIR) principles (Ouyang et al., 2021; Sharma et al., 2021).The users can explore scRNA-seq data in hESCNeuroDiffscRNA and plot high resolution figures of their genes of interest under seven different tabs (Figures S7A–S7H; Table S7). This includes exploration of 1) Gene expression UMAPS as illustrated for *POU5F1* and *NTRK1*; 2) gene co-expression analysis, here shown for *PHC1/PHC2* and *NEUROG1/NTRK1*; 3) different gene and cluster expression configurations, such as heatmaps, violin-, box-, proportion- and bubble plots. The platform also allows for correlation with other published gene expression datasets (Figure S7H).

**Figure 7:**
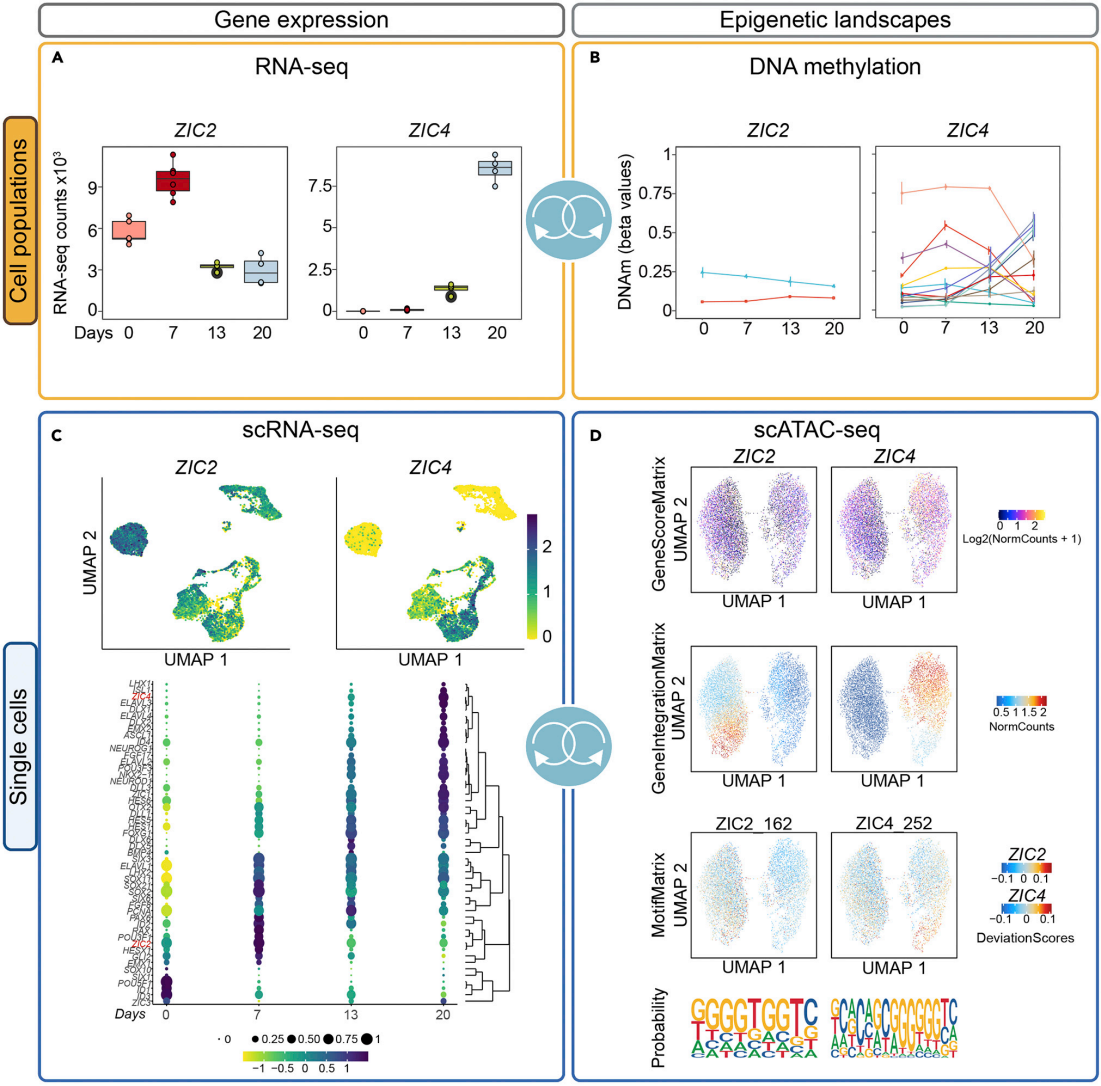
An example of the possibilities and potential applications of the 4D data showcasing the *ZIC2* and *ZIC4* genes (A) Normalized global gene expression counts for *ZIC2* and *ZIC4* from Days 0, 7, 13 and 20. (B) Significant CpGs in gene loci *ZIC2* and *ZIC4* (derived from MORE) for Days 0, 7, 13 and 20. DNAm is represented as mean +/− standard deviation. (C) Representative UMAPs showing cluster specific and differentiation-driven gene expression across all four timepoints for *ZIC2* and *ZIC4*. A bubble plot representing gene expression and hierarchical clustering of TFs highly relevant to neuronal differentiation across Days 0, 7, 13, and 20. (D) The upper UMAPS represent inferred gene scores of the openness of the *ZIC2* and *ZIC4* gene loci. In middle UMAPs the gene integration shows correlation of gene expression with chromatin opening of the *ZIC2* and *ZIC4* gene loci. The lower UMAPs represent motif footprinting demonstrating preferential opening in different cell clusters for ZIC2 and ZIC4 with the representative sequence logos identified in accessible regions in the dataset below. See also Figure S7.

To exemplify the web-interface, we focus on TFs *ZIC2* and *ZIC4* (Figure 7). ZIC proteins are known for their role in proliferation and early neuronal developmental processes (Al-Naama et al., 2020; Aruga and Millen, 2018). Global expression analysis shows that *ZIC2* is present at Day 0 and peaks at Day 7, whereas expression of *ZIC4*, mostly undetectable at early timepoints, appears at Day 13 and peaks at Day 20 (Figure 7A). DNAm levels at the CpGs in the *ZIC2* locus were stable, whereas DNAm of 13 CpGs in the *ZIC4* locus were positively or negatively correlated with gene expression across differentiation (Figure 7B). Differential expression of *ZIC2* and *ZIC4* across individual cells at Day 0, 7, 13 and 20 in UMAPs (Figure 7C) can be compared and correlated with TFs important in the neuronal differentiation protocol (Figure 7C).

The scATAC-seq data can be explored in “hESC Neuro Differentiation scATAC seq” (hESCNeuroDiffscATAC) (Figures S7I–S7N; Table S7). Users can visualize chromatin accessibility, motif enrichment or integration of scATAC-seq with scRNA-seq in different UMAPs (Figure S7I). The webtool enables investigation of gene score and motif matrix, showing the representative sequence logo calculated from open regions. (gene examples at Figure S7J). Chromatin opening can be explored across the genome for different clusters, as shown for *SOX2* and *POU5F1* or in different heatmaps (Figures S7K–S7N; Table S7). Pseudotime trajectories or peak-to-gene linkage can be viewed as heatmaps, which define linked chromatin opening peaks with promoters of expressed genes (Granja et al., 2021) and may deduce enhancer promoter interactions (Baek and Lee, 2020). Gene score and gene integration analysis showed that *ZIC2* and *ZIC4* were active in different cells (Figure 7D) whereas their footprints and representative sequence logos were identified. Our 4D approach highlights the epigenetic regulation and gene expression of these genes using this neuronal differentiation protocol.

## Discussion

Here we present comprehensive multi-omics analyses to characterize a 2D neuronal differentiation protocol from pluripotent hESCs towards a ventrally committed, telencephalic population of progenitors, mature and immature neurons. We assessed stage-to-stage transition using ddPCR and immunofluorescence imaging and used bulk RNA-seq and scRNA-seq to validate gene expression and cell populations over time. The scATAC-seq and DNAm analyses further characterized the epigenetic and gene regulatory landscapes. We have identified cis-regulatory elements and DNA binding TFs that regulate neuronal differentiation gene expression programs.

The deconvolution of early human neuronal differentiation at the level of molecular regulation provides insight to an otherwise inaccessible developmental window. Animal models are valuable, but evidence shows that the human neocortex develops under the effect of additional mechanism (Massimo and Long, 2021; Pinson and Huttner, 2021; Xing et al., 2021). Thus, neuronal differentiation studies from PSCs provide an alternative method to characterize developmental transcriptome trajectories and the roles of specific genes in human brain formation and patterning.

To specify the patterning and cell maturation identities, we followed up the trajectories of major TFs (O’Leary and Sahara, 2008). Ventral telencephalic markers, such as *EMX2* and *ASCL1*, were already expressed at the end of Stage II, whereas dorsal markers such as *EMX1* and *NEUROG2* were absent. Absence of expression of *HOXB2*, *PAX7*, and *GBX2*, confirmed that self-patterning after neural induction, had no effect on lineage commitment.

GO analyses revealed stage dependent enrichment of biological processes correlated to neurogenesis, pattern specification, signaling and neurotransmitter regulation, migration, synaptic organization and neuronal maturation. The DNAm analyses showed alternating, stage-dependent changes for various patterning genes and TFs important to neurogenesis. In most developmentally regulated genes involved in neuronal lineage commitment, DNAm levels decreased on transcriptional activation and increased for genes becoming repressed during the time of differentiation. We also observed that sometimes downregulation of gene expression in the self-patterning stage might intersect the upregulation of gene expression seen both in stage I of neural induction of hESCs and during maturation at stage III. This could be because of the combined effect of non-CpG and CpG DNAm implicated in the regulation of RNA splicing in ESCs and neurons, respectively (Ball et al., 2009; Laurent et al., 2010). Non-CpG DNAm accumulates in neurons during synaptogenesis and synaptic pruning (Lister et al., 2013), and CpH DNAm is associated with transcriptional repression (Luo et al., 2016; Xie et al., 2012). Whether and how CpH DNAm plays a role in self patterning following the LSX induction is not known, and future studies are needed to explore this. Furthermore, the *in vitro* model for DNAm changes presented here is advantageous for neuropharmacological studies. Whether these changes can be translated to distinct early developmental events, cannot be ascertained. The direction of causality of epigenetic regulation for early brain development can, however, further correlate *in vitro* models to sets of open cis-regulatory elements and the regulation of TF-centered networks. The identification of common DNAm modification sites and chromatin openness regions may present candidate loci for future studies of early human development and may advance translational studies of the impact of drugs used early in human pregnancy.

The effect of loss of pluripotency towards neuralization, irrespective of the intermediate timepoints, was investigated through an integrative analysis of the scATAC- and scRNA-seq. We used ArchR (Granja et al., 2021) for this analysis as this pipeline was flexible for small scATAC-seq and scRNA-seq datasets. The juxtaposition of the transcriptome to the regulatory elements in ESCs and differentiating Day 20 cells allowed us to infer neuronal gene regulatory network information.

We identified linked sets of genes and putative CREs unique to hESCs and Day 20 that enabled us to infer epigenome regulation. Less than 2.7% of the putative CREs identified in Day 0 or Day 20 overlapped with the annotated ENSEMBL enhancers. However, our functional assessment of the potential CREs suggests that we have identified new enhancers specific for neuronal differentiation. A greater number of expression linked putative CREs was identified for Day 20 than in the more homogenous hESCs, and the functional annotations for the linked analysis correlates with the BPs from differential analysis of RNA-seq and DNAm. Our multi-omics approach showed that the *in vitro* 2D neuronal differentiation protocol recapitulates stages of neuronal progenitor proliferation and specification. Thousands of transient chromatin accessible regions linked to expressed genes have been mapped in early human brain development (de la Torre-Ubieta et al., 2018; Trevino et al., 2020, 2021; Ziffra et al., 2021). Using the same correlation based approach for scATAC-seq and scRNA-seq integration, we were able to compare CRE-linked genes that were active in Day 20 with CRE-linked genes from human brain tissue, at weeks 16 to 24 after conception (Trevino et al., 2021). A total of 68% linked genes from the early human brain single-cell pseudotime integration overlapped with the Day 20 CRE-linked genes. Moreover, we show that the genomic location of some of the putative CREs for neuronal genes overlapped with active early human brain enhancers assessed in transgenic mouse embryos (Visel et al., 2013). Future work need to establish the functional role of the predicted Day 20 CREs in neuronal differentiation. Analyses of datasets from studies such as ours could define the epigenetic regulatory programs preceding and regulating gene expression during cortical development. The causal direction of epigenetic regulation in early brain development was further corroborated with the *in vitro* differentiation via sets of open CREs and the regulation of neuronal TF-centered networks. However, only 10% of regulatory TFs identified in putative CREs in the same pseudotime developmental brain program overlapped with Day 20 mapped regulatory TFs (Table S5) (Trevino et al., 2021). Buenostro et al. have reported observations that epigenetic regulation are found to precede transcription (Ma et al., 2020). We suggest that some of the detected chromatin accessible regions in Day 20 cells define gene expression for future cellular states. Our comprehensive gene expression and epigenetic regulation network is a resource for future studies including, but not restricted to, the effect of specific disease variants or drugs in early brain physiology and development.

## Strengths of study

Although numerous studies have used the LSX cocktail for neural induction, to our knowledge this is the first study that has shared all scRNA-seq and scATAC-seq data in such transparent and interactive format. Thus the strength of this study is the high quality data and the presentation of our single-cell data in two visualization tools, ShinyCell and in-house developed ShinyArchR.UiO (Ouyang et al., 2021; Sharma et al., 2021), that are openly available for users. These tools allow the users to explore candidate genes and utilize a comprehensive set of functionalities, beyond the analysis presented. These tools enable insight into the molecular and structural partners of stage-specific markers and time-stamped TFs, their transcriptional regulation and cell cluster identities. Programming scripts for data analysis are made available and can be easily customized for further studies and the incorporation of other data.

### Limitations of the study

The protocol described is an *in vitro* study, and as such, it is difficult to know how well it reflects the *in vivo* dynamics. Furthermore, this study has several limitations at the level of neuronal subtype characterization, as we did not assess neuropeptide diversity or secretion and the membrane electrochemical and electrophysiological maturation properties were not evaluated. Protein quantification or intracellular localization of markers and trajectories were also beyond the scope of this multi-omics characterization. A limitation of scATAC-seq is the relatively low genome-per-cell coverage, thus open chromatin regions relevant for the individual cell or cell populations may have been missed. Lastly, we were unable to accurately classify the cells in the G0 phase. This challenge is not a limitation of this particular study, highlighting the need to better understand G0 dynamics. The outcome would be the design of tools that can accurately predict the cell cycle phase in differentiating and differentiated neurons.

In conclusion, in this study we describe the characterization of a 2D neuronal differentiation protocol where the unparalleled power of multi-omics was used to decipher fate specification. We assessed the functional regulation of transcription factors and developmentally regulated genes, from loss of pluripotency towards neuronal differentiation. Integration of scATAC-seq and scRNA-seq provide invaluable insight on the complexity of fate decisions and enable other researchers to fine-tune future studies. Finally, the reader has access to the single-cell sequencing data in two searchable, user-friendly webtools to visualize intra- and inter- time point and cell cluster regulation, interactively.

## STAR★Methods

### Key resources table

**Table:** 

REAGENT or RESOURCE	SOURCE	IDENTIFIER
**Antibodies**		

OCT4 (1/100)	Santa Cruz Biotech	Cat# sc-5279; RRID:AB_628051
β3-tubulin (1/500)	Santa Cruz Biotech	Cat# sc-80005; RRID:AB_2210816
OTX2 (1/40)	R&D Systems	Cat# AF1979; RRID:AB_2157172
SOX2 (1/250)	Santa Cruz Biotech	Cat# sc-365823; RRID:AB_10842165
PAX6 (1/500)	Santa Cruz Biotech	Cat# sc-81649; RRID:AB_1127044
NESTIN (1/500)	Santa Cruz Biotech	Cat# sc-23927;RRID:AB_627994
Donkey Anti-Goat IgG H&L (Alexa Fluor® 555) (1/500)	Abcam	ab150130
Donkey Anti-Mouse IgG H&L (Alexa Fluor® 555) (1/500)	Abcam	Cat# ab150110; RRID:AB_2783637
Alexa Fluor® 488 AffiniPure Donkey Anti-Rabbit IgG (H + L) (1/250)	Jackson ImmunoResearch	711-545-152

**Chemicals, peptides, and recombinant proteins**		

Geltrex™ LDEV-Free, hESC-Qualified, Reduced Growth Factor Basement Membrane Matrix	ThermoFisher	A1413302
KnockOut™ DMEM	ThermoFisher	10829018
PBS, no calcium, no magnesium	ThermoFisher / GIBCO	14190
Dimethyl-sulfoxide, DMSO	Sigma-Aldrich/ Merck	D8418
Accutase™ Cell Detachment Solution	STEMCELL Technologies	7920
UltraPure 0.5 M EDTA, pH 8.0	ThermoFisher	15575020
RHO/ROCK Pathway Inhibitor Y-27632	STEMCELL Technologies	SCM075
Essential 8™ Medium	ThermoFisher	A1517001
Poly-L-ornithine hydrobromide	Sigma-Aldrich/ Merck	P3655
Fibronectin (Bovine Protein, Plasma)	ThermoFisher	33010018
N2 supplement (100X)	ThermoFisher	17502048
Advanced DMEM/F-12	ThermoFisher	12634028
GlutaMAX™ Supplement	GIBCO/ ThermoFisher	35050061
Penicillin Streptomycin (10,000 U/mL)	ThermoFisher	15140122
LDN-193189	STEMCELL Technologies	72148
SB 431542 (hydrate)	Sigma-Aldrich / Merck	S4317
XAV939	STEMCELL Technologies	72674
B-27™ Supplement (50X), serum free	ThermoFisher	17504044
Recombinant Human FGF basic	Peprotech	100-18B
Recombinant Human EGF, Animal-Free	Peprotech	AF-100-15
Invitrogen™ ProLong™ Gold Antifade Mountant with DAPI	Fisher Scientific/ Invitrogen	P36931
Paraformaldehyde	Sigma-Aldrich/ Merck	158127
Triton X-100	ThermoFisher	11332481001
Tween-20	Sigma-Aldrich/ Merck	P1379
Normal-Horse-Serum-Blocking-Solution	BioNordica/ Vectorlabs	S-2000-20
Bovine Serum Albumin	Sigma-Aldrich/ Merck	A2153

**Critical commercial assays**		

Countess™ Cell Counting Chamber Slides	ThermoFisher	C10312
RNeasy Mini Kit	Qiagen	74106
RNAse-Free DNase Set	Qiagen	79254
RNA/DNA purification kit	Norgen Biotek Corp.	298–48700
RNase-Free DNase I Kit	Norgen Biotek Corp.	298–25720
Qubit™ RNA BR Assay Kit	ThermoFisher/Invitrogen	Q10211
QuantiTect Reverse Transcription Kit	Qiagen	205311
ddPCR Supermix for Probes (no dUTP)	BioRad	186–3024
Droplet Generation Oil for Probes	BioRad	186–3005
TruSeq Stranded mRNA Library Prep Kit	Illumina	20020595
IDT for Illumina – TruSeq RNA UD Indexes	Illumina	20022371
NovaSeq 6000 S1 Reagent Kit v1.5 (200 cycles)	Illumina	20028318
Infinium MethylationEPIC BeadChip Kit (96 samples)	Illumina	WG-317-1003
30 mm MACS SmartStrainers	Miltenyi Biotech	130-110-915
Chromium Single Cell 3′ Library & Gel Bead Kit v3	10x Genomics	1000075
Chromium i7 Multiplex Kit	10x Genomics	120262
NextSeq 500/550 High Output Kit (150 Cycles)	Illumina	20024907
Next GEM Chip H Single Cell Kit	10x Genomics	1000162
Next GEM Single Cell ATAC Library & Gel Bead Kit v1.1	10x Genomics	1000176
Chromium i7 Multiplex Kit N Set A	10x Genomics	1000084
NovaSeq 6000 SP Reagent Kit (100 cycles)	Illumina	20028401

**Deposited data**		

RNA-seq, DNAm, Infinium Methylation EPIC, scRNA-seq & scATAC-seq	This paper	NCBI GEO: GSE192858 (Subseries GSE192854, GSE192855, GSE192856, GSE192857)

**Experimental models: Cell lines**		

Human embryonic cells, HS360, 46XY	Stockholms Medicinska Biobank / Sweden	HS360

**Oligonucleotides**		

*POU5F1*	ThermoFisher/TaqMan™	Hs00999632_g1
*SOX2*	ThermoFisher/TaqMan™	Hs01053049_s1
*NANOG*	ThermoFisher/TaqMan™	Hs04399610_g1
*NES*	ThermoFisher/TaqMan™	Hs04187831_g1
*FOXG1*	ThermoFisher/TaqMan™	Hs01850784_s1
*TUBB3*	ThermoFisher/TaqMan™	Hs00801390_s1
*MAP2*	ThermoFisher/TaqMan™	Hs00258900_m1
*PAX6*	ThermoFisher/TaqMan™	Hs00240871_m1
*OTX2*	ThermoFisher/TaqMan™	Hs00222238_m1
*VIM*	ThermoFisher/TaqMan™	Hs00958111_m1
*NEUROD1*	ThermoFisher/TaqMan™	Hs01922995_s1
*RPL30*	ThermoFisher/TaqMan™	Hs00265497_m1
*RAF1* F: tgggaaatagaagccagtgaa R: cctttaggatctttactgcaacatc	Eurofins	Probe 56 4688538001

**Software and algorithms**		

R Programming language	(R Core Team, 2019)	https://www.r-project.org/
ArchR1.0.1	(Granja et al., 2021)	https://www.archrproject.com
Seurat Version 4	(Hao et al., 2021; Stuart et al., 2019)	https://github.com/satijalab/seurat
Signac	(Stuart et al., 2021)	https://satijalab.org/signac/
BSgenome1.58.0	(Pagès, 2020)	https://rdrr.io/bioc/BSgenome/
ShinyCell	(Ouyang et al., 2021)	https://github.com/SGDDNB/ShinyCell
ShinyArchR.UiO	(Sharma et al., 2021)	https://github.com/EskelandLab/ShinyArchRUiO
CytoTRACE R package (v0.3.3)	(Gulati et al., 2020)	https://cytotrace.stanford.edu
10x Genomics Cell Ranger-Count and 10x Genomics Cell Ranger-Count ATAC	10x genomics	https://www.10xgenomics.com
FIJI	(Schindelin et al., 2012)	https://imagej.net/software/fiji/
BSgenome.Hsapiens.UCSC.hg38	https://doi.org/10.18129/B9.bioc.BSgenome.Hsapiens.UCSC.hg38 (The Bioconductor Dev Team, 2021)	https://bioconductor.org/packages/release/data/annotation/html/BSgenome.Hsapiens.UCSC.hg38.html
EnsDb.Hsapiens.v86	https://doi.org/10.18129/B9.bioc.EnsDb.Hsapiens.v86 (Rainer, 2017)	https://bioconductor.org/packages/release/data/annotation/html/EnsDb.Hsapiens.v86.html
clustree	(Zappia and Oshlack, 2018)	https://cran.r-project.org/web/packages/clustree/vignettes/clustree.html#references
scater	(McCarthy et al., 2017)	https://bioconductor.org/packages/release/bioc/html/scater.html
DEseq2	(Love et al., 2014)	https://bioconductor.org/packages/release/bioc/html/DESeq2.html
GSEA	(Subramanian et al., 2005)	https://www.gsea-msigdb.org/gsea/index.jsp
Minfi	(Aryee et al., 2014)	https://www.bioconductor.org/packages/release/bioc/html/minfi.html
Limma	(Ritchie et al., 2015)	https://bioconductor.org/packages/release/bioc/html/limma.html
missMethyl	(Phipson et al., 2016)	https://bioconductor.org/packages/release/bioc/html/missMethyl.html
MORE	(Conesa, 2018)	https://github.com/ConesaLab/MORE
SingleR	(Aran et al., 2019)	https://github.com/dviraran/SingleR
Harmony	(Korsunsky et al., 2019)	https://github.com/immunogenomics/harmony
GREAT	(McLean et al., 2010)	http://great.stanford.edu/public/html/
Intervene	(Khan and Mathelier, 2017)	https://github.com/asntech/intervene-shiny
gprofiler	(Kolberg et al., 2020)	https://biit.cs.ut.ee/gprofiler/gost
MACS2	(Feng et al., 2012)	https://pypi.org/project/MACS2/ https://github.com/macs3-project/MACS
refdata-cellranger-atac-hg38-version refdata-	10x genomics	https://support.10xgenomics.com/single-cell-atac/software/downloads/
JASPAR	(Bryne et al., 2008)	https://bioconductor.org/packages/release/data/annotation/html/JASPAR2020.html http://jaspar.genereg.net
Single Cell Experiment	(Amezquita et al., 2020)	https://bioconductor.org/packages/release/bioc/html/SingleCellExperiment.html
Escape	https://doi.org/10.18129/B9.bioc.escape; (Borcherding and Andrews, 2022)	https://github.com/ncborcherding/escape
ggseqlogo	(Wagih, 2017)	https://CRAN.R-project.org/package=ggseqlogo
TFBSTools	https://doi.org/10.18129/B9.bioc.TFBSTools	https://bioconductor.org/packages/release/bioc/html/TFBSTools.html
uwot	https://github.com/jlmelville/uwot	https://CRAN.R-project.org/package=uwot
viridisLite	(Garnier et al., 2021)	https://cran.r-project.org/web/packages/viridisLite/index.html
ShinyGO	(Ge et al., 2020)	http://bioinformatics.sdstate.edu/go/
Python3	(Van Rossum and Drake, 2009)	https://www.python.org/downloads/
10x Genomics Loupe Browser	10x genomics	https://www.10xgenomics.com
ComplexHeatmap	(Gu, 2022; Gu et al., 2016)	https://jokergoo.github.io/ComplexHeatmap-reference/book/
ggplot2	(Wickham, 2009)	https://cran.r-project.org/web/packages/ggplot2/index.html
igraph	igraph	https://igraph.org
IRanges	(Lawrence et al., 2013)	https://bioconductor.org/packages/release/bioc/html/IRanges.html
Reticulate	Tomasz Kalinowski, R Studio	https://CRAN.R-project.org/package=reticulate
tidyverse	(Wickham et al., 2019)	https://www.tidyverse.org/packages/
ggpubr	(Kassambara, 2020)	https://cran.r-project.org/web/packages/ggpubr/index.html
SARTools	(Varet et al., 2016)	https://github.com/PF2-pasteur-fr/SARTools
pheatmap	Raivo Kolde, 2019	https://CRAN.R-project.org/package=pheatmap
lluminaHumanMethylationEPICmanifest	Hansen, 2016	https://bioconductor.org/packages/release/data/annotation/html/IlluminaHumanMethylationEPICmanifest.html
IlluminaHumanMethylationEPICanno.ilm10b5.hg38	EPIC annotation 1.0 B5	https://github.com/achilleasNP/IlluminaHumanMethylationEPICanno.ilm10b5.hg38
Rstudio	RStudio Team	https://www.rstudio.com/
Shiny single-cell tools for visualisation of datasets.	This paper	Custom scripts for computational analysis are available at https://github.com/EskelandLab/scNeuronaldiff. Single-cell data can be explored in webtools “hESC Neuronal Differentiation scRNA-seq” and “hESC Neuro Differentiation scATAC seq” at https://cancell.medisin.uio.no/

### Resource availability

#### Lead contact

Further information and requests for resources and reagents should be directed to and will be fulfilled by the lead contact Ragnhild Eskeland (Ragnhild.Eskeland@medisin.uio.no).

#### Materials availability

This study did not generate new unique reagents.

### Experimental model and subject details

#### Human embryonic stem cell culture and maintenance and neuronal differentiation protocol

The hESC line HS360 (Stockholm Medical Biobank, Sweden, KIe009-A) (Main et al., 2020; Ström et al., 2010) was used in this study. hESCs were maintained in Essential 8™ Medium (Thermo Fisher), in feeder-free conditions on Geltrex Matrix solution (Thermo Fisher) pre-coated culture plates and media was replaced daily. The full description of hESC cultivation and a step-by-step description of the 2D neuronal differentiation protocol, all stock solutions and dilution recipes, coating instructions and times, critical points of the protocol, a Day-by-Day timeline of high-resolution brightfield images, immunocytochemistry, qRT-PCR marker genes for HS360 are available at STAR Protocols (Samara et al., 2022). Brightfield images were acquired on an EVOS® FL Cell Imaging System AMF4300 (Thermo Fisher).

### Methods details

#### Immunofluorescence analysis

In brief, cells grown on 13mm glass coverslips, were washed once and fixed in 4% paraformaldehyde for 15 min at room temperature (RT). After 3 washes, the cells were permeabilized with 0.3% Triton X-100 (Thermo Fisher) in blocking buffer containing 2% BSA (Sigma-Aldrich) and 0.01% Tween in 1×PBS for 30minat RT, washed 3 times, and blocked with 10% horse serum for 30 min. Primary antibodies were diluted (as in KRT) in 1×PBS containing 0.03% Triton X-100, and coverslips were incubated overnight at 4°C. Next, coverslips were equilibrated at RT for 2 h and washed 3 times. The secondary antibodies were diluted (see key resources table) in 0.01% Tween-20 (Sigma-Aldrich) and 0.1% horse serum (BioNordika) in 1×PBS, and coverslips were incubated for 1 h at RT. The coverslips were washed 3 times and mounted on microscope slides using the ProLong™ Gold Antifade Mountant containing DAPI (Fisher Scientific) to counterstain cell nuclei. Washing steps lasted 15 min and used 1×PBS. Images were obtained with a DeltaVision high resolution widefield microscope (GE Life Sciences, USA) using the Resolve 3D software and 100×1.45NA oil objective and processed using the open-source software Fiji (Schindelin et al., 2012).

#### DNA/RNA isolation

Genomic DNA and total RNA were isolated by direct lysis in the culture well followed by column-based isolation using RNA/DNA purification kit (Norgen Biotek). The RNase-Free DNase I Kit (Norgen Biotek) was applied for on-column removal of genomic DNA contamination from RNA isolates. Three RNA isolates were processed using RNeasy Mini Kit (Qiagen) followed by DNase-treatment using RNAse-Free DNase Set (Qiagen). All isolations were done according to the manufacturer’s instructions. Nucleic acid quantification was performed using Qubit (Thermo Fisher Scientific), purity was measured using Nanodrop 2000 (Thermo Fisher Scientific), whereas RNA and DNA integrity was assessed using 2100 Bioanalyzer (Agilent Technologies) and 4200 TapeStation (Agilent Technologies), respectively.

#### Droplet digital RT-PCR and RNA expression analysis

Reverse transcription of total RNA was performed using QuantiTect Reverse Transcription Kit (Qiagen). Subsequent ddPCR reactions were set up using ddPCR Supermix for Probes (No dUTP) (BioRad) and Taqman assays (Thermo Fisher) or Universal Probes (Roche) in combination with target primers (Eurofins) as outlined in KRT/Oligonucleotides. Droplets for droplet PCR amplification were generated using the QX200 Droplet Generator (BioRad). Data acquisition and primary analysis was done using the QX200 Droplet Reader (BioRad) and QuantaSoft software (BioRad). All steps were performed according to the manufacturer’s instructions. To calculate the number of target copies per ng RNA input, samples were normalized using *RPL30* and *RAF1* as normalization genes (Coulter, 2018). Results were visualized in R using the tidyverse package (Wickham et al., 2019). Statistical comparisons for ddRT-PCR and RNA expression analysis were performed in R using t-test in ggpubr package v.0.4.0 (Kassambara, 2020).

#### Global RNA-seq

The sequencing library was prepared with TruSeq Stranded mRNA Library Prep (Illumina) according to manufacturer’s instructions. The 19 libraries were pooled at equimolar concentrations and sequenced on an Illumina NovaSeq 6000 S1 flow cell (Illumina) with 100 bp paired end reads. The quality of sequencing reads was assessed using BBMap (Bushnell, 2014), and adapter sequences and low-quality reads were removed. The sequencing reads were then mapped to the GRCh38.p5 index using HISAT2 (Kim et al., 2015, p. 2). Mapped paired end reads were counted to protein coding genes using featureCounts (Liao et al., 2014). Differential expression analysis was conducted in R version 3.5.1 (R Core Team, 2019) using SARTools v.1.6.8 (Varet et al., 2016) and the DESeq2 v.1.22.1 (Love et al., 2014), and genes were considered significantly differentially expressed with an FDR <0.01. Normalized counts were visualized using the tidyverse package v.1.3.0 (Wickham et al., 2019). The heatmaps were generated using the pheatmap package version 1.0.12 (Kolde, 2019). The Wald test was used to calculate p-values and Benjamini-Hochberg was used to correct for multiple testing. The gene ontology (GO) analysis of a ranked list of differential expressed genes were performed using GSEA software (Subramanian et al., 2005) looking at biological process (BP) terms.

#### Illumina EPIC array

DNA methylation status of 22 samples was assessed using the Infinium MethylationEPIC BeadChip v.1.0_B3 (Illumina). Quality control and pre-processing of the raw data was performed in R using Minfi v.1.36.0 (Aryee et al., 2014). No samples were removed because of poor quality (detection p values > 0.05). Background correction was performed using NOOB method (Triche et al., 2013) and β values (ratio of methylated signal divided by the sum of the methylated and unmethylated signal) were normalized using functional normalization (Fortin et al., 2014). Probes with unreliable measurements (detection p values > 0.01) (n = 8,818) and cross-reactive probes (Chen et al., 2013, p. 450) (n = 43,256) were then removed, resulting in a final dataset consisting of 814,112 probes and 22 samples. Probes were annotated with Illumina Human Methylation EPIC annotation 1.0 B5 (hg38). Differential methylation (DM) analysis was performed on the M values (log2 of the β values) using the Limma package (Ritchie et al., 2015), and CpGs were considered significantly differentially methylated with an FDR <0.01. GO analysis was performed using top 10% DM CpGs (DMCs) as input to GOMETH in the missMethyl package version 1.24.0 (Phipson et al., 2016) for BP terms.

### Integration of RNA-seq and DNA methylation data

Data from matching DNA and RNA samples (extracted from the same wells, n = 16) were subsetted to undergo statistical integration. Multi-Omics Regulation (MORE) (Conesa, 2018) was used to identify CpGs that regulate gene expression by applying Generalized Linear Models: normalized counts for differentially expressed genes (from DEseq2) were used as the response variable, CpG M-values (from Minfi) and experimental covariates (Day) were used as predictors. First, CpGs with low variability were filtered and multicollinearity was reduced by grouping highly correlated CpGs. Variable selection was then performed with Elastic Net regression and stepwise (two-ways backward) regression. CpGs were considered to significantly regulate gene expression when the regression coefficient p-value was <0.05. Significant CpG regulators of gene expression were visualized using the Tidyverse package (Wickham et al., 2019) using beta values (n = 22) and normalized counts (n = 19) from all samples.

#### Collection of cells and scRNA-seq

HS360 hESCs were differentiated in two separate time-course experiments. Cells harvested on Days 0, 7, 13 and 20 were washed twice in wells with 1xPBS and detached using Accutase (STEMCELL Technologies) at 37°C for 7 min. Cells were triturated 10–15 times to separate into single cells and transferred to centrifuge tubes containing the appropriate base media with 0.05% BSA (Sigma-Aldrich). Counts were performed using Countess II FL Cell Counter (Thermo Fisher Scientific), cells were centrifuged at 300x g for 5 min and the supernatant was discarded. Cell pellets were then resuspended in base medium containing 0.05% BSA and cell aggregates were filtered out using MACS SmartStrainers (Miltenyi). The cells were recounted and processed within 1 h on the 10x Chromium controller (10x Genomics). Approximately 2,300 cells were loaded per channel on the Chromium Chip B (10x Genomics) to give an estimated recovery of 1,400 cells. The Chromium Single Cell 3′ Library & Gel Bead Kit v3 (10x Genomics) and Chromium i7 Multiplex Kit (10x Genomics) were used to generate scRNA-seq libraries, according to the manufacturer’s instructions. Libraries from 16 samples were pooled together based on molarity and sequenced on a NextSeq 550 (Illumina) with 28 cycles for read 1, 8 cycles for the I7 index and 91 cycles for read 2. For the second sequencing run, libraries were pooled again based on the number of recovered cells to give a similar number of reads per cell for each sample (33,000–44,000 reads/cell).

#### scRNA-seq data analysis

The Cell Ranger 3.1.0 Gene Expression pipeline (10x Genomics) was used to demultiplex the raw base-call files and convert them into FASTQ files. The FASTQ files were aligned to the GRCh38–3.0.0 human reference genome, and Cell Ranger count was used with default parameters for computing read counts for Days 0, 7, 13 and 20.

We utilized Harmony for batch correction after merging and performing log normalization on the two replicates (Korsunsky et al., 2019). We aggregated sequenced replicates for each day into single datasets using Cell Ranger Aggr command. The Seurat Package v.4.0. (Hao et al., 2021) was used to perform quality control and normalization on the count matrices obtained after the aggregation. The gene count per cell, UMI count per cell and percentage of mitochondrial and ribosomal transcripts were computed using the functions of the Seurat package. Low quality cells expressing few genes (less than 200) were excluded from the downstream analysis. Genes expressed in less than three cells were removed. Duplicates, dead cells and cells with greater than 5 median absolute deviations (MADs) for mitochondrial reads were filtered out (McCarthy et al., 2017). We used the MAD-based definition of outliers, using the Isoutlier function of scater package version 1.0.4, to remove putative low-quality cells from the dataset. For normalization we used scTRANSFORM to better understand cell to cell heterogeneity after performing cell cycle regression analysis (Hafemeister and Satija, 2019; Tirosh et al., 2016). Counts were adjusted for cell-specific sampling (‘normalized’) using the scTRANSFORM function with regression of cell cycle genes and mitochondrial content. To cluster the cells, we used resolution of 0.55, obtained by determining the optimum number of clusters (cell grouped together sharing similar expression profiles) in the dataset using the Clustree R package (Zappia and Oshlack, 2018) (Figures S4B and S4C). SingleR (Aran et al., 2019) was used to annotate the cells against two different reference data sets. Global RNA-seqdata from Human Primary Cell Atlas was accessed using the celldex (Aran et al., 2019), and scRNA-seq data from a Human Brain dataset (La Manno et al., 2016) was accessed using the scRNAseq R package . Cell types with < 15 cells annotated were excluded from the plots (Figures S4F and 4D). FindMarkers from the Seurat R package was used to perform differential expression analysis between days.

#### Cell-cycle state assessment using scRNA-seq data

The cell cycle state assessment was performed using a gene set and scoring system previously reported (Tirosh et al., 2016). The S and G2M scores were calculated based on 43 S phase-specific genes and 54 G2 or M phase-specific genes and the Seurat package Cell cycle scoring function was used for calculation of actual scores.

#### Clustering and dimensionality reduction

The Resolution for finding clusters were computed using scClustviz SNN- based clustering (Innes and Bader, 2018). Then, principal component analysis was performed using RunPCA function of the Seurat package. For UMAP visualization, clusters were identified using FindNeighbors and FindClusters Seurat function using resolution 0.55, followed by the RunUMAP function across samples with the same parameters. UMAP preserves aspects of global structure in larger datasets and was therefore preferred for visualization over t-SNE (Becht et al., 2019). We used FindMarkers or FindAllMarkers functions to compute differentially expressed genes between the clusters.

#### CytoTRACE

We utilized CytoTRACE (Cellular (Cyto) Trajectory Reconstruction Analysis) (Gulati et al., 2020) with gene counts for all datasets (Merged Days 0, 7, 13 and 20 and Day 0 and Day 20) for prediction of differentiation state of cells from scRNA-seq data. In short, CytoTRACE leverages single cell gene counts, covariant gene expression and local neighbourhoods of transcriptionally similar cells to predict ordered differentiation states from scRNA-seq data. CytoTRACE uses smoothing steps within the dataset to remove confounding factors associated with direct comparison of genes expressed by each cell in cross-study differences in depth and sensitivity.

#### scATAC-seq Library Preparation and sequencing

Cells from HS360 hESCs were differentiated in one time-course experiment, and at Day 0 and Day 20, the cells were washed twice with 1xPBS and detached to single cell suspension by application of Accutase (STEMCELL Technologies) at 37°C for 7 min. The detached cells were washed with appropriate base media with added 0.04% BSA (Sigma-Aldrich) and filtered using MACS SmartStrainers (Miltenyi Biotech) to remove cell aggregates. Nuclei isolation was done according to the 10x Genomics protocol CG000169 (Rev D) using 2 min of incubation in lysis buffer diluted to 0.1x and 0.5x for Day 0 and Day 20 cells, respectively. We used the Countess II FL Cell Counter (Thermo Fisher Scientific) to quantify nuclei and confirm complete lysis and microscopy to confirm high nuclei quality. Nuclei were further processed on the 10x Chromium controller (10x Genomics) using Next GEM Chip H Single Cell Kit (10x Genomics), Next GEM Single Cell ATAC Library & Gel Bead Kit v1.1 (10 x Genomics) and Chromium i7 Multiplex Kit N Set A (10x Genomics) according to the Next GEM Single Cell ATAC Reagent Kits v1.1 User Guide (CG000209, Rev C). The targeted nuclei recovery was 5,000 nuclei per sample. The resulting 4 sample libraries were sequenced on a NovaSeq Sp flow cell (Illumina) with 50 cycles for read 1, 8 cycles for the i7 index read, 16 cycles for the i5 index read and 49 cycles for read 2.

#### scATAC sequencing analysis

Cell Ranger ATAC version 1.2.0 with reference genome GRCh38–1.2.0 was used to pre-process scATAC-seq raw sequencing data into FASTQ files. Single cell accessibility counts for the cells were generated from reads using the ‘cellranger-atac count’ pipeline. Reference genome HG38 used for alignment and generation of single-cell accessibility counts was obtained from the 10x Genomics (https://support.10xgenomics.com/single-cell-atac/software/downloads/). Downstream analysis of the scATAC-seq data was performed using the R package ArchR v1.0.1 (Granja et al., 2021). A tile matrix of 500-bp bins was constructed after quality control, removal of low-quality cells and doublet removal using the *doubletfinder* function of ArchR. The ArchR Project contained the filtered cells that had a TSS enrichment below 3 and <1000 fragments. ArchR has implemented Harmony for batch correction (Korsunsky et al., 2019). A layered dimensionality reduction approach utilizing Latent Semantic Indexing (LSI) and Singular Value Decomposition (SVD) applied on Genome-wide tile matrix. Uniform Manifold approximation and projection (UMAP) was performed to visualize data in 2D space. Louvain Clustering methods implemented in R package Seurat (Stuart et al., 2019) was used for clustering of the single-cell accessibility profiles. We also obtained links (peak2genelinks) between gene TSS and putative CRE’s to expression profiles of genes from ArchR. The peak2genelinks pair represents enhanced gene interactions. We correlated the putative CREs to a list of Human Regulatory Regions (GRCh38.p13) from Ensembl Regulatory Build (Zerbino et al., 2015). The active enhancer sequences for *ASCL1* and *ID4* were extracted from the VISTA Enhancer Browser (https://enhancer.lbl.gov/) (Visel et al., 2007) for visualization with putative D20 CREs in UCSC genome browser (Kent et al., 2002). We next performed GREAT analysis on D0 and D20 potential CREs on each timepoint separately to identify biological processes associated with peak2genelinks clustered together with 5 k-means (McLean et al., 2010). Positive TF regulators were identified, given JASPAR motif and accessibility is correlated with their gene activity either on a gene score matrix (gene expression) or on gene integration matrix (integrated scATAC-seq with scRNA-seq experiments, storing matched scRNA profiles).

#### Single-cell ATAC sequencing analysis

Cross platform linkage of scRNA-Seq data with a scATAC-seq data unconstrained and constrained integration aims to align cells from scATAC-seq and scRNA-seq experiments by combining them both together. A constrained integration can improve the quality of cross-platform alignment by limiting the alignment search space based on prior knowledge of the celltype. ArchR performs an unconstrained integration to determine preliminary cluster identities, followed by a refined constrained integration based on this prior knowledge (Granja et al., 2021). As part of the scATAC-seq and scRNA-seq integration, the GeneIntegration matrix containing linked gene expression data is added to HDF5 formatted arrow files. The GeneIntegrationMatrix enabled us to compare the linked gene expression with the gene score estimates of gene expression. The genes associated with known and new marker genes related to pluripotency, neuronal differentiation, and cell cycle of single cell RNA-seq were constrained and used as new labels of integrated clusters.

#### Pseudo-bulk replicates and peak calling

Using ArchR’s addReproduciblePeakSet() function utilizing MACS2 for peak calling, we created a reproducible MACS2-derived merged peak set (Granja et al., 2021; Zhang et al., 2008). The new peak matrix was added to the ArchR project using AddpeakMatrix function on MACS2-derived merged peak set. AddMarkerFeatures() was used to compute unique peaks for each cluster or group of clusters. Using the markerHeatmap() function we visualized marker peaks in heatmaps and the Plotbrowsertrack function to visualize the tracks. Motif enrichment was used to predict what TFs may mediate the binding events that create the accessible chromatin sites. By examining the differential peak opening in the different cell populations, we can identify motifs that have a high level of enrichment. A motif’s presence in a peak set was determined using the AddmotifAnnotation function. PeakAnnotation was performed using peakAnnoEnrichment() on Jaspar2020 (Bryne et al., 2008). To compute representative seq logo for the motifs from position weight matrix we used the ggSeqlogo package (Wagih, 2017).

#### ChromVAR deviations enrichment with ArchR

ChromVAR deviations for each single cell were calculated using the “addDeviationsMatrix” function (Granja et al., 2021). To visualize transcription factor (TF) footprinting we used the “plotFootprints” function with the normalization method “subtract” which subtracts the Tn5 bias from the ATAC-seq footprint.

#### TF footprinting

We used the ArchR addCoAccessibility() function to store the peak co-accessibility information and the getCoAccessibility function to retrieve it at the loop (Granja et al., 2021). Peak co-accessibility is a correlation of accessibility between two peaks across many cells. We performed peak-coaccessibility analysis to predict regulatory interactions in the scATAC-seq dataset and the integration with scRNA-seq data such that upstream CRE activity can be predicted through peak-to-gene linkage analyses. We also used the Peak-to-gene linkage analysis to plot Peak2GeneHeatmap, containing two side-by-side heatmaps, one pertaining to scATAC-seq gene score data and the other to scRNA-seq gene expression data. In addition, we analyzed the enrichment of TF binding motifs using the peakAnnoEnrichment function implemented in ArchR (Granja et al., 2021).

### Pseudo-time trajectory analysis

We constructed a cellular trajectory spanning constrained integrated clusters R0, R2, R7, R9 to R12, using the addTrajectory function implemented in ArchR on GeneScore, GeneIntegration, Peak and Tilematrix (Granja et al., 2021).

### Quantification and statistical analysis

Statistical analyses were performed in R version 4.1.2 (R Core Team, 2019) using SARTools v.1.6.8 (Varet et al., 2016), DESeq2 v.1.22.1 (Love et al., 2014), Limma (Ritchie et al., 2015), ArchR (Granja et al., 2021), Seurat (Hao et al., 2021; Stuart et al., 2019), MORE (Conesa, 2018) and ggpubr package v.0.4.0 (Kassambara, 2020). Details are described in the relevant methods sections above.

## Data Availability

• All original code has been deposited at https://github.com/EskelandLab/scNeuronaldiff. DOIs are listed in the key resources table. • The single-cell RNA-seq/ATAC-seq, RNA-seq and DNA-methylation data reported in this study cannot be deposited in a public repository because the data could be potentially traced back to a single embryo and the donor. To request access, contact the lead author and the Stockholm Medical Biobank. It may be required to establish a Personal Data processing (PDP) Agreement and/or Data Transfer Agreement (DTA) according to General Data Protection Regulation (GDPR). • Processed datasets have been deposited at NCBI’s GEO Super series GSE192858. Accession numbers are listed in the key resources table. Single-cell data are shared for visualization in two open access webtools at https://cancell.medisin.uio.no: https://cancell.medisin.uio.no/scrna/hescneurodiff/ and https://cancell.medisin.uio.no/scatac/hescneurodiff.archr/

## Supplemental information

Document S1. Figures S1–S7 and Tables S1 and S7

Table S2. GSEA for all timepoints in the protocol, related to Figs. 2 and S2

Table S3. Overview of significant CpG regulators of genes, related to Figs. 3C and S3F and S3G

Table S4. Quality control, cell metadata, and cluster information, related to Figs. 4 and S4 (A) Cell Ranger QC output. (B) Cell numbers and cluster populations (%) per timepoint. (C–G) Differential gene expression analysis between Days 0, 7, 13 and 20. (H) Percent overlap significantly differentially expressed genes between RNA-seq and scRNA-seq, related to Fig. 4.

Table S5. Inferred peak to gene links, TF motif and GO-term enrichment of Day 20 (A) Inferred peak to gene links Day 20. (B) GOProfiler GO terms for Day 20 K- mean clusters 1–5. (C–G) Gene ontology annotation for peak2genelink k-mean clusters 1–5 for Day 20 using Great Tool. (H) Comparison of linked genes (P2G links) of Day 20 with Trevino et al., 2021, Cell Table S3. (I) TF motif enrichment in Day 20 five interaction clusters (k-mean clusters 1–5). (J) TF motif enrichment in Day 20 five interaction clusters (k-mean clusters 1–5) compared to Trevino et al., 2021, Cell TF motif enrichment (Trevino Fig. 3G), related to Fig. 6.

Table S6. Inferred peak to gene links, TF motif and GO-term enrichment of Day 0 (A) Inferred peak to gene links Day 0; (B) GOProfiler GO terms for Day 0 K-mean clusters 1–5; (C–G) Gene ontology annotation for peak2genelink K-mean clusters 1–5 for Day 0 using Great Tool; TF motif enrichment in Day 0 five interaction clusters (k-mean clusters 1–5), related to Fig. S6.

## References

[bib1] Al-Naama N., Mackeh R., Kino T. (2020). C2H2-Type zinc finger proteins in brain development, neurodevelopmental, and other neuropsychiatric disorders: systematic literature-based analysis. Front. Neurol..

[bib2] Amezquita R.A., Lun A.T.L., Becht E., Carey V.J., Carpp L.N., Geistlinger L., Marini F., Rue-Albrecht K., Risso D., Soneson C. (2020). Orchestrating single-cell analysis with Bioconductor. Nat. Methods.

[bib3] Amiri A., Coppola G., Scuderi S., Wu F., Roychowdhury T., Liu F., Pochareddy S., Shin Y., Safi A., Song L., Zhu Y., Sousa A.M.M., Gerstein M., Crawford G.E., Sestan N., Abyzov A., Vaccarino F.M., PsychENCODE Consortium (2018). Transcriptome and epigenome landscape of human cortical development modeled in organoids. Science.

[bib4] Arai Y., Pulvers J.N., Haffner C., Schilling B., Nüsslein I., Calegari F., Huttner W.B. (2011). Neural stem and progenitor cells shorten S-phase on commitment to neuron production. Nat. Commun..

[bib5] Aran D., Looney A.P., Liu L., Wu E., Fong V., Hsu A., Chak S., Naikawadi R.P., Wolters P.J., Abate A.R. (2019). Reference-based analysis of lung single-cell sequencing reveals a transitional profibrotic macrophage. Nat. Immunol..

[bib6] Aruga J., Millen K.J., Aruga J. (2018). Zic Family: Evolution, Development and Disease, Advances in Experimental Medicine and Biology.

[bib7] Aryee M.J., Jaffe A.E., Corrada-Bravo H., Ladd-Acosta C., Feinberg A.P., Hansen K.D., Irizarry R.A. (2014). Minfi: a flexible and comprehensive Bioconductor package for the analysis of Infinium DNA methylation microarrays. Bioinformatics.

[bib8] Baek S., Lee I. (2020). Single-cell ATAC sequencing analysis: from data preprocessing to hypothesis generation. Comput. Struct. Biotechnol. J..

[bib9] Ball M.P., Li J.B., Gao Y., Lee J.-H., LeProust E.M., Park I.-H., Xie B., Daley G.Q., Church G.M. (2009). Targeted and genome-scale strategies reveal gene-body methylation signatures in human cells. Nat. Biotechnol..

[bib10] Becht E., McInnes L., Healy J., Dutertre C.-A., Kwok I.W.H., Ng L.G., Ginhoux F., Newell E.W. (2019). Dimensionality reduction for visualizing single-cell data using UMAP. Nat. Biotechnol..

[bib11] Becker K.A., Ghule P.N., Therrien J.A., Lian J.B., Stein J.L., van Wijnen A.J., Stein G.S. (2006). Self-renewal of human embryonic stem cells is supported by a shortened G1 cell cycle phase. J. Cell. Physiol..

[bib12] Beyer T.A., Weiss A., Khomchuk Y., Huang K., Ogunjimi A.A., Varelas X., Wrana J.L. (2013). Switch enhancers interpret TGF-β and hippo signaling to control cell fate in human embryonic stem cells. Cell Rep..

[bib13] Borcherding N., Andrews J. (2022). escape: easy single cell analysis platform for enrichment.

[bib14] Boward B., Wu T., Dalton S. (2016). Concise Review: control of cell fate through cell cycle and pluripotency networks. Stem Cell..

[bib15] Bryne J.C., Valen E., Tang M.-H.E., Marstrand T., Winther O., da Piedade I., Krogh A., Lenhard B., Sandelin A. (2008). JASPAR, the open access database of transcription factor-binding profiles: new content and tools in the 2008 update. Nucleic Acids Res..

[bib16] Bushnell B. (2014).

[bib17] Cakir B., Xiang Y., Tanaka Y., Kural M.H., Parent M., Kang Y.-J., Chapeton K., Patterson B., Yuan Y., He C.-S. (2019). Engineering of human brain organoids with a functional vascular-like system. Nat. Methods.

[bib18] Chavali V.R.M., Haider N., Rathi S., Vrathasha V., Alapati T., He J., Gill K., Nikonov R., Duong T.T., McDougald D.S. (2020). Dual SMAD inhibition and Wnt inhibition enable efficient and reproducible differentiations of induced pluripotent stem cells into retinal ganglion cells. Sci. Rep..

[bib19] Chen L., Tong Q., Chen X., Jiang P., Yu H., Zhao Q., Sun L., Liu C., Gu B., Zheng Y. (2021). PHC1 maintains pluripotency by organizing genome-wide chromatin interactions of the Nanog locus. Nat. Commun..

[bib20] Chen Y.a., Lemire M., Choufani S., Butcher D.T., Grafodatskaya D., Zanke B.W., Gallinger S., Hudson T.J., Weksberg R. (2013). Discovery of cross-reactive probes and polymorphic CpGs in the Illumina Infinium HumanMethylation450 microarray. Epigenetics.

[bib21] Chiu W.T., Charney Le R., Blitz I.L., Fish M.B., Li Y., Biesinger J., Xie X., Cho K.W.Y. (2014). Genome-wide view of TGFβ/Foxh1 regulation of the early mesendoderm program. Development.

[bib22] Chou S.-J., Tole S. (2019). Lhx2, an evolutionarily conserved, multifunctional regulator of forebrain development. Brain Res..

[bib23] Colasante G., Simonet J.C., Calogero R., Crispi S., Sessa A., Cho G., Golden J.A., Broccoli V. (2015). ARX regulates cortical intermediate progenitor cell expansion and upper layer neuron formation through repression of Cdkn1c. Cereb. Cortex.

[bib24] Conesa A. (2018).

[bib25] Coulter S.J. (2018). Mitigation of the effect of variability in digital PCR assays through use of duplexed reference assays for normalization. Biotechniques.

[bib26] de la Torre-Ubieta L., Stein J.L., Won H., Opland C.K., Liang D., Lu D., Geschwind D.H. (2018). The dynamic landscape of open chromatin during human cortical neurogenesis. Cell.

[bib27] Eze U.C., Bhaduri A., Haeussler M., Nowakowski T.J., Kriegstein A.R. (2021). Single-cell atlas of early human brain development highlights heterogeneity of human neuroepithelial cells and early radial glia. Nat. Neurosci..

[bib28] Fedorova V., Vanova T., Elrefae L., Pospisil J., Petrasova M., Kolajova V., Hudacova Z., Baniariova J., Barak M., Peskova L. (2019). Differentiation of neural rosettes from human pluripotent stem cells in vitro is sequentially regulated on a molecular level and accomplished by the mechanism reminiscent of secondary neurulation. Stem Cell Res..

[bib29] Feng J., Liu T., Qin B., Zhang Y., Liu X.S. (2012). Identifying ChIP-seq enrichment using MACS. Nat. Protoc..

[bib30] Fortin J.-P., Labbe A., Lemire M., Zanke B.W., Hudson T.J., Fertig E.J., Greenwood C.M., Hansen K.D. (2014). Functional normalization of 450k methylation array data improves replication in large cancer studies. Genome Biol..

[bib31] Garnier S., Ross N., Camargo A.P., Rudis boB., Woo K. (2021). sjmgarnier/viridisLite: CRAN release v0.4.0.

[bib32] Ge S.X., Jung D., Yao R. (2020). ShinyGO: a graphical gene-set enrichment tool for animals and plants. Bioinformatics.

[bib33] Granja J.M., Corces M.R., Pierce S.E., Bagdatli S.T., Choudhry H., Chang H.Y., Greenleaf W.J. (2021). ArchR is a scalable software package for integrative single-cell chromatin accessibility analysis. Nat. Genet..

[bib34] Gu Z. (2022). Complex heatmap visualization. iMeta.

[bib35] Gu Z., Eils R., Schlesner M. (2016). Complex heatmaps reveal patterns and correlations in multidimensional genomic data. Bioinformatics.

[bib36] Gulati G.S., Sikandar S.S., Wesche D.J., Manjunath A., Bharadwaj A., Berger M.J., Ilagan F., Kuo A.H., Hsieh R.W., Cai S. (2020). Single-cell transcriptional diversity is a hallmark of developmental potential. Science.

[bib37] Hafemeister C., Satija R. (2019). Normalization and variance stabilization of single-cell RNA-seq data using regularized negative binomial regression. Genome Biol..

[bib138] Hansen K.D. (2016). IlluminaHumanMethylationEPICmanifest: Manifest for Illumina’s EPIC methylation arrays.

[bib38] Hao Y., Hao S., Andersen-Nissen E., Mauck W.M., Zheng S., Butler A., Lee M.J., Wilk A.J., Darby C., Zager M. (2021). Integrated analysis of multimodal single-cell data. Cell.

[bib39] Hao Y., Tang S., Yuan Y., Liu R., Chen Q. (2019). Roles of FGF8 subfamily in embryogenesis and oral-maxillofacial diseases (Review). Int. J. Oncol..

[bib40] Hasenpusch-Theil K., West S., Kelman A., Kozic Z., Horrocks S., McMahon A.P., Price D.J., Mason J.O., Theil T. (2018). Gli3 controls the onset of cortical neurogenesis by regulating the radial glial cell cycle through Cdk6 expression. Development.

[bib41] Hong S.-H., Lee J.-H., Lee J.B., Ji J., Bhatia M. (2011). ID1 and ID3 represent conserved negative regulators of human embryonic and induced pluripotent stem cell hematopoiesis. J. Cell Sci..

[bib42] Hu Q.-D., Ang B.-T., Karsak M., Hu W.-P., Cui X.-Y., Duka T., Takeda Y., Chia W., Sankar N., Ng Y.-K. (2003). F3/Contactin acts as a functional ligand for Notch during oligodendrocyte maturation. Cell.

[bib43] Iida H., Furukawa Y., Teramoto M., Suzuki H., Takemoto T., Uchikawa M., Kondoh H. (2020). Sox2 gene regulation via the D1 enhancer in embryonic neural tube and neural crest by the combined action of SOX2 and ZIC2. Gene Cell..

[bib44] Ikeda K., Ookawara S., Sato S., Ando Z.i., Kageyama R., Kawakami K. (2007). Six1 is essential for early neurogenesis in the development of olfactory epithelium. Dev. Biol..

[bib45] Innes B.T., Bader G.D. (2018). scClustViz - single-cell RNAseq cluster assessment and visualization. F1000Res.

[bib46] Iyer A.K., Miller N.L.G., Yip K., Tran B.H., Mellon P.L. (2010). Enhancers of GnRH transcription embedded in an upstream gene use homeodomain proteins to specify hypothalamic expression. Mol. Endocrinol..

[bib47] Kassambara A. (2020).

[bib48] Kent W.J., Sugnet C.W., Furey T.S., Roskin K.M., Pringle T.H., Zahler A.M., Haussler D. (2002). The human genome browser at UCSC. Genome Res..

[bib49] Khan A., Mathelier A. (2017). Intervene: a tool for intersection and visualization of multiple gene or genomic region sets. BMC Bioinf..

[bib50] Kim D., Langmead B., Salzberg S.L. (2015). HISAT: a fast spliced aligner with low memory requirements. Nat. Methods.

[bib51] Kirkeby A., Grealish S., Wolf D.A., Nelander J., Wood J., Lundblad M., Lindvall O., Parmar M. (2012). Generation of regionally specified neural progenitors and functional neurons from human embryonic stem cells under defined conditions. Cell Rep..

[bib52] Kolberg L., Raudvere U., Kuzmin I., Vilo J., Peterson H. (2020).

[bib53] Kolde R. (2019).

[bib54] Korsunsky I., Millard N., Fan J., Slowikowski K., Zhang F., Wei K., Baglaenko Y., Brenner M., Loh P.R., Raychaudhuri S. (2019). Fast, sensitive and accurate integration of single-cell data with Harmony. Nat. Methods.

[bib55] Kurtz A., Zimmer A., Schnütgen F., Brüning G., Spener F., Müller T. (1994). The expression pattern of a novel gene encoding brain-fatty acid binding protein correlates with neuronal and glial cell development. Development.

[bib56] La Manno G., Gyllborg D., Codeluppi S., Nishimura K., Salto C., Zeisel A., Borm L.E., Stott S.R.W., Toledo E.M., Villaescusa J.C. (2016). Molecular diversity of midbrain development in mouse, human, and stem cells. Cell.

[bib57] Laukoter S., Beattie R., Pauler F.M., Amberg N., Nakayama K.I., Hippenmeyer S. (2020). Imprinted Cdkn1c genomic locus cell-autonomously promotes cell survival in cerebral cortex development. Nat. Commun..

[bib58] Laurent L., Wong E., Li G., Huynh T., Tsirigos A., Ong C.T., Low H.M., Kin Sung K.W., Rigoutsos I., Loring J., Wei C.-L. (2010). Dynamic changes in the human methylome during differentiation. Genome Res..

[bib59] Lawrence M., Huber W., Pagès H., Aboyoun P., Carlson M., Gentleman R., Morgan M.T., Carey V.J. (2013). Software for computing and annotating genomic ranges. PLoS Comput. Biol..

[bib60] Lee S.-J., Jung Y.H., Oh S.Y., Yong M.S., Ryu J.M., Han H.J. (2014). Netrin-1 induces MMP-12-dependent E-cadherin degradation via the distinct activation of PKCα and FAK/fyn in promoting mesenchymal stem cell motility. Stem Cells Dev..

[bib61] Li X., Sun L., Jin Y. (2008). Identification of karyopherin-alpha 2 as an Oct4 associated protein. J. Genet. Genomics.

[bib62] Liao Y., Smyth G.K., Shi W. (2014). featureCounts: an efficient general purpose program for assigning sequence reads to genomic features. Bioinformatics.

[bib63] Lister R., Mukamel E.A., Nery J.R., Urich M., Puddifoot C.A., Johnson N.D., Lucero J., Huang Y., Dwork A.J., Schultz M.D. (2013). Global epigenomic reconfiguration during mammalian brain development. Science.

[bib64] Lister R., Pelizzola M., Dowen R.H., Hawkins R.D., Hon G., Tonti-Filippini J., Nery J.R., Lee L., Ye Z., Ngo Q.-M. (2009). Human DNA methylomes at base resolution show widespread epigenomic differences. Nature.

[bib65] Liu L., Michowski W., Kolodziejczyk A., Sicinski P. (2019). The cell cycle in stem cell proliferation, pluripotency and differentiation. Nat. Cell Biol..

[bib66] Love M.I., Huber W., Anders S. (2014). Moderated estimation of fold change and dispersion for RNA-seq data with DESeq2. Genome Biol..

[bib67] Luo C., Lancaster M.A., Castanon R., Nery J.R., Knoblich J.A., Ecker J.R. (2016). Cerebral organoids recapitulate epigenomic signatures of the human fetal brain. Cell Rep..

[bib68] Ma S., Zhang B., LaFave L.M., Earl A.S., Chiang Z., Hu Y., Ding J., Brack A., Kartha V.K., Tay T. (2020). Chromatin potential identified by shared single-cell profiling of RNA and chromatin. Cell.

[bib69] Main H., Hedenskog M., Acharya G., Hovatta O., Lanner F. (2020). Karolinska institutet human embryonic stem cell bank. Stem Cell Res..

[bib70] Major T., Powers A., Tabar V. (2016). Derivation of telencephalic oligodendrocyte progenitors from human pluripotent stem cells. Curr. Protoc. Stem Cell Biol..

[bib71] Maksimovic J., Oshlack A., Phipson B. (2021). Gene set enrichment analysis for genome-wide DNA methylation data. Genome Biol..

[bib72] Markenscoff-Papadimitriou E., Whalen S., Przytycki P., Thomas R., Binyameen F., Nowakowski T.J., Kriegstein A.R., Sanders S.J., State M.W., Pollard K.S., Rubenstein J.L. (2020). A chromatin accessibility atlas of the developing human telencephalon. Cell.

[bib73] Maroof A.M., Keros S., Tyson J.A., Ying S.-W., Ganat Y.M., Merkle F.T., Liu B., Goulburn A., Stanley E.G., Elefanty A.G. (2013). Directed differentiation and functional maturation of cortical interneurons from human embryonic stem cells. Cell Stem Cell.

[bib74] Massimo M., Long K.R. (2021). Orchestrating human neocortex development across the scales; from micro to macro. Semin. Cell Dev. Biol..

[bib75] McCarthy D.J., Campbell K.R., Lun A.T.L., Wills Q.F. (2017). Scater: pre-processing, quality control, normalization and visualization of single-cell RNA-seq data in R. Bioinformatics.

[bib76] McLean C.Y., Bristor D., Hiller M., Clarke S.L., Schaar B.T., Lowe C.B., Wenger A.M., Bejerano G. (2010). GREAT improves functional interpretation of cis-regulatory regions. Nat. Biotechnol..

[bib77] Mistri T.K., Devasia A.G., Chu L.T., Ng W.P., Halbritter F., Colby D., Martynoga B., Tomlinson S.R., Chambers I., Robson P., Wohland T. (2015). Selective influence of Sox2 on POU transcription factor binding in embryonic and neural stem cells. EMBO Rep..

[bib78] Nadarajah B., Parnavelas J.G. (2002). Modes of neuronal migration in the developing cerebral cortex. Nat. Rev. Neurosci..

[bib79] O'Leary D.D., Sahara S. (2008). Genetic regulation of arealization of the neocortex. Curr. Opin. Neurobiol..

[bib80] Ohashi M., Korsakova E., Allen D., Lee P., Fu K., Vargas B.S., Cinkornpumin J., Salas C., Park J.C., Germanguz I. (2018). Loss of MECP2 leads to activation of P53 and neuronal senescence. Stem Cell Rep..

[bib81] Ouyang J.F., Kamaraj U.S., Cao E.Y., Rackham O.J.L. (2021). ShinyCell: simple and sharable visualization of single-cell gene expression data. Bioinformatics.

[bib82] Pagès H. (2020). https://rdrr.io/bioc/BSgenome/.

[bib83] Pang T., Atefy R., Sheen V. (2008). Malformations of cortical development. Neurol..

[bib84] Pfister A.S., Tanneberger K., Schambony A., Behrens J. (2012). Amer2 protein is a novel negative regulator of wnt/β-catenin signaling involved in neuroectodermal patterning. J. Biol. Chem..

[bib85] Phipson B., Maksimovic J., Oshlack A. (2016). missMethyl: an R package for analyzing data from Illumina’s HumanMethylation450 platform. Bioinformatics.

[bib86] Pinson A., Huttner W.B. (2021). Neocortex expansion in development and evolution-from genes to progenitor cell biology. Curr. Opin. Cell Biol..

[bib87] Piper M., Barry G., Harvey T.J., McLeay R., Smith A.G., Harris L., Mason S., Stringer B.W., Day B.W., Wray N.R. (2014). NFIB-mediated repression of the epigenetic factor Ezh2 regulates cortical development. J. Neurosci..

[bib88] R Core Team (2019).

[bib89] Rainer J. (2017). EnsDb.Hsapiens.v86. Bioconductor. http://bioconductor.org/packages/EnsDb.Hsapiens.v86/.

[bib90] Reilly S.K., Yin J., Ayoub A.E., Emera D., Leng J., Cotney J., Sarro R., Rakic P., Noonan J.P. (2015). Evolutionary changes in promoter and enhancer activity during human corticogenesis. Science.

[bib91] Riemens R.J.M., van den Hove D.L.A., Esteller M., Delgado-Morales R. (2018). Directing neuronal cell fate in vitro: achievements and challenges. Prog. Neurobiol..

[bib92] Ritchie M.E., Phipson B., Wu D., Hu Y., Law C.W., Shi W., Smyth G.K. (2015). Limma powers differential expression analyses for RNA-sequencing and microarray studies. Nucleic Acids Res..

[bib93] Samara A., Spildrejorde M., Sharma A., Falck M., Leithaug M., Modafferi S., Bjørnstad P.M., Acharya G., Gervin K., Lyle R., Eskeland R. (2022). A multi-omics approach to visualize early neuronal differentiation in 4D. bioRxiv.

[bib94] Schindelin J., Arganda-Carreras I., Frise E., Kaynig V., Longair M., Pietzsch T., Preibisch S., Rueden C., Saalfeld S., Schmid B. (2012). Fiji: an open-source platform for biological-image analysis. Nat. Methods.

[bib95] Schlosser G. (2014). Early embryonic specification of vertebrate cranial placodes. Wiley Interdiscip. Rev. Dev. Biol..

[bib96] Schultz M.D., He Y., Whitaker J.W., Hariharan M., Mukamel E.A., Leung D., Rajagopal N., Nery J.R., Urich M.A., Chen H. (2015). Human body epigenome maps reveal noncanonical DNA methylation variation. Nature.

[bib97] Shah S.N., Kerr C., Cope L., Zambidis E., Liu C., Hillion J., Belton A., Huso D.L., Resar L.M.S. (2012). HMGA1 reprograms somatic cells into pluripotent stem cells by inducing stem cell transcriptional networks. PLoS One.

[bib98] Sharma A., Akshay A., Rogne M., Eskeland R. (2021). ShinyArchR.UiO: user-friendly, integrative and open-source tool for visualization of single-cell ATAC-seq data using ArchR. Bioinformatics.

[bib99] Smith Z.D., Meissner A. (2013). DNA methylation: roles in mammalian development. Nat. Rev. Genet..

[bib100] Soufi A., Dalton S. (2016). Cycling through developmental decisions: how cell cycle dynamics control pluripotency, differentiation and reprogramming. Development.

[bib101] Spalice A., Parisi P., Nicita F., Pizzardi G., Del Balzo F., Iannetti P. (2009). Neuronal migration disorders: clinical, neuroradiologic and genetics aspects. Acta Paediatr..

[bib102] Stricker S.H., Götz M. (2018). DNA-methylation: master or slave of neural fate decisions?. Front. Neurosci..

[bib103] Ström S., Holm F., Bergström R., Strömberg A.-M., Hovatta O. (2010). Derivation of 30 human embryonic stem cell lines--improving the quality. In Vitro Cell. Dev. Biol. Anim..

[bib104] Stuart T., Butler A., Hoffman P., Hafemeister C., Papalexi E., Mauck W.M., Hao Y., Stoeckius M., Smibert P., Satija R. (2019). Comprehensive integration of single-cell data. Cell.

[bib105] Stuart T., Srivastava A., Madad S., Lareau C.A., Satija R. (2021). Single-cell chromatin state analysis with Signac. Nat. Methods.

[bib106] Subramanian A., Tamayo P., Mootha V.K., Mukherjee S., Ebert B.L., Gillette M.A., Paulovich A., Pomeroy S.L., Golub T.R., Lander E.S., Mesirov J.P. (2005). Gene set enrichment analysis: a knowledge-based approach for interpreting genome-wide expression profiles. Proc. Natl. Acad. Sci. USA.

[bib107] Sun J., Yang J., Miao X., Loh H.H., Pei D., Zheng H. (2021). Proteins in DNA methylation and their role in neural stem cell proliferation and differentiation. Cell Regen..

[bib108] Tang K., Peng G., Qiao Y., Song L., Jing N. (2015). Intrinsic regulations in neural fate commitment. Dev. Growth Differ..

[bib109] Tchieu J., Zimmer B., Fattahi F., Amin S., Zeltner N., Chen S., Studer L. (2017). A modular platform for differentiation of human PSCs into all major ectodermal lineages. Cell Stem Cell.

[bib110] The Bioconductor Dev Team (2021). BSgenome.Hsapiens.UCSC.hg38. Bioconductor. http://bioconductor.org/packages/BSgenome.Hsapiens.UCSC.hg38/.

[bib111] Tirosh I., Izar B., Prakadan S.M., Wadsworth M.H., Treacy D., Trombetta J.J., Rotem A., Rodman C., Lian C., Murphy G. (2016). Dissecting the multicellular ecosystem of metastatic melanoma by single-cell RNA-seq. Science.

[bib112] Trevino A.E., Müller F., Andersen J., Sundaram L., Kathiria A., Shcherbina A., Farh K., Chang H.Y., Pașca A.M., Kundaje A. (2021). Chromatin and gene-regulatory dynamics of the developing human cerebral cortex at single-cell resolution. Cell.

[bib113] Trevino A.E., Sinnott-Armstrong N., Andersen J., Yoon S.-J., Huber N., Pritchard J.K., Chang H.Y., Greenleaf W.J., Pașca S.P. (2020). Chromatin accessibility dynamics in a model of human forebrain development. Science.

[bib114] Triche T.J., Weisenberger D.J., Van Den Berg D., Laird P.W., Siegmund K.D. (2013). Low-level processing of Illumina Infinium DNA methylation BeadArrays. Nucleic Acids Res..

[bib115] van der Raadt J., van Gestel S.H.C., Nadif Kasri N., Albers C.A. (2019). ONECUT transcription factors induce neuronal characteristics and remodel chromatin accessibility. Nucleic Acids Res..

[bib116] Van Rossum G., Drake F.L. (2009).

[bib117] Varet H., Brillet-Guéguen L., Coppée J.Y., Dillies M.-A. (2016). SARTools: a DESeq2- and EdgeR-based R pipeline for comprehensive differential analysis of RNA-seq data. PLoS One.

[bib118] Verrotti A., Spalice A., Ursitti F., Papetti L., Mariani R., Castronovo A., Mastrangelo M., Iannetti P. (2010). New trends in neuronal migration disorders. Eur. J. Paediatr. Neurol..

[bib119] Visel A., Minovitsky S., Dubchak I., Pennacchio L.A. (2007). VISTA Enhancer Browser—a database of tissue-specific human enhancers. Nucleic Acids Res..

[bib120] Visel A., Taher L., Girgis H., May D., Golonzhka O., Hoch R.V., McKinsey G.L., Pattabiraman K., Silberberg S.N., Blow M.J. (2013). A high-resolution enhancer atlas of the developing telencephalon. Cell.

[bib121] Wagih O. (2017). ggseqlogo: a versatile R package for drawing sequence logos. Bioinformatics.

[bib122] Wang H., Wang X., Xu X., Kyba M., Cooney A.J. (2016). Germ cell nuclear factor (GCNF) represses Oct4 expression and globally modulates gene expression in human embryonic stem (hES) cells. J. Biol. Chem..

[bib123] Wang H., Xiao Z., Zheng J., Wu J., Hu X.-L., Yang X., Shen Q. (2019). ZEB1 represses neural differentiation and cooperates with CTBP2 to dynamically regulate cell migration during neocortex development. Cell Rep..

[bib124] Wang Q., Zhang Y., Wang M., Song W.-M., Shen Q., McKenzie A., Choi I., Zhou X., Pan P.-Y., Yue Z., Zhang B. (2019). The landscape of multiscale transcriptomic networks and key regulators in Parkinson’s disease. Nat. Commun..

[bib125] Wickham H. (2009). ggplot2: Elegant Graphics for Data Analysis, Use R! Springer-Verlag, New York.

[bib126] Wickham H., Averick M., Bryan J., Chang W., McGowan L., François R., Grolemund G., Hayes A., Henry L., Hester J. (2019). Welcome to the tidyverse. J. Open Source Softw..

[bib127] Xie W., Barr C.L., Kim A., Yue F., Lee A.Y., Eubanks J., Dempster E.L., Ren B. (2012). Base-resolution analyses of sequence and parent-of-origin dependent DNA methylation in the mouse genome. Cell.

[bib128] Xie W., Schultz M.D., Lister R., Hou Z., Rajagopal N., Ray P., Whitaker J.W., Tian S., Hawkins R.D., Leung D. (2013). Epigenomic analysis of multilineage differentiation of human embryonic stem cells. Cell.

[bib129] Xing L., Wilsch-Bräuninger M., Huttner W.B. (2021). How neural stem cells contribute to neocortex development. Biochem. Soc. Trans..

[bib130] Yao B., Christian K.M., He C., Jin P., Ming G.L., Song H. (2016). Epigenetic mechanisms in neurogenesis. Nat. Rev. Neurosci..

[bib131] Zappia L., Oshlack A. (2018). Clustering trees: a visualization for evaluating clusterings at multiple resolutions. GigaScience.

[bib132] Zerbino D.R., Wilder S.P., Johnson N., Juettemann T., Flicek P.R. (2015). The Ensembl regulatory Build. Genome Biol..

[bib133] Zhang S., Bell E., Zhi H., Brown S., Imran S.A.M., Azuara V., Cui W. (2019). OCT4 and PAX6 determine the dual function of SOX2 in human ESCs as a key pluripotent or neural factor. Stem Cell Res. Ther..

[bib134] Zhang Y., Liu T., Meyer C.A., Eeckhoute J., Johnson D.S., Bernstein B.E., Nusbaum C., Myers R.M., Brown M., Li W., Liu X.S. (2008). Model-based analysis of ChIP-seq (MACS). Genome Biol..

[bib135] Zheng X., Boyer L., Jin M., Mertens J., Kim Y., Ma L., Ma L., Hamm M., Gage F.H., Hunter T. (2016). Metabolic reprogramming during neuronal differentiation from aerobic glycolysis to neuronal oxidative phosphorylation. Elife.

[bib136] Zhu Q., Song L., Peng G., Sun N., Chen J., Zhang T., Sheng N., Tang W., Qian C., Qiao Y. (2014). The transcription factor Pou3f1 promotes neural fate commitment via activation of neural lineage genes and inhibition of external signaling pathways. Elife.

[bib137] Ziffra R.S., Kim C.N., Ross J.M., Wilfert A., Turner T.N., Haeussler M., Casella A.M., Przytycki P.F., Keough K.C., Shin D. (2021). Single-cell epigenomics reveals mechanisms of human cortical development. Nature.

