# Supplementary material for: A multi-omics approach to visualize early neuronal differentiation from hESCs in 4D
Source: iScience. 2022 Oct 4;25(11):105279. doi: 10.1016/j.isci.2022.105279 (PMC9593815; doi:10.1016/j.isci.2022.105279)
Supplement: Document S1. Figures S1–S7 and Tables S1 and S7 [file mmc1.pdf]

iScience, Volume *25*

Supplemental information

A multi-omics approach to visualize early neuronal differentiation from hESCs in 4D

Athina Samara, Mari Spildrejorde, Ankush Sharma, Martin Falck, Magnus Leithaug, Stefania Modafferi, Pål Marius Bjørnstad, Ganesh Acharya, Kristina Gervin, Robert Lyle, and Ragnhild Eskeland

Supplemental Information

A multi-omics approach to visualize early neuronal differentiation from hESCs in 4D

Athina Samara[$,1,2,#], Mari Spildrejorde[$,3,4,5], Ankush Sharma[$,6,7,8,*], Martin Falck[3,8,&], Magnus Leithaug[4,§], Stefania Modafferi[4,+], Pål Marius Bjørnstad[4], Ganesh Acharya[9,10], Kristina Gervin[3,11,12], Robert Lyle[3,4,13,#] and Ragnhild Eskeland[3,7,14,#]

[$] These authors contributed equally

# Co-corresponding authors

[1]Division of Clinical Paediatrics, Department of Women's and Children's Health, Karolinska Institutet, Sweden
[2]Astrid Lindgren Children's Hospital Karolinska University Hospital, Stockholm, Sweden
[3]PharmaTox Strategic Research Initiative, Faculty of Mathematics and Natural Sciences, University of Oslo, Norway
[4]Department of Medical Genetics, Oslo University Hospital and University of Oslo, Norway
[5]Institute of Clinical Medicine, Faculty of Medicine, University of Oslo, Oslo, Norway.
[6]Department of Informatics, University of Oslo, Norway
[7] Department of Molecular Medicine, Institute of Basic Medical Sciences, Faculty of Medicine, University of Oslo, Oslo, Norway
[8]Department of Biosciences, University of Oslo, Norway
[9] Division of Obstetrics and Gynecology, Department of Clinical Science, Intervention and Technology (CLINTEC), Karolinska Institutet, Alfred Nobels Allé 8, SE-14152, Stockholm, Sweden.
[10]Center for Fetal Medicine, Karolinska University Hospital Huddinge, SE-14186 Stockholm, Sweden.
[11]Pharmacoepidemiology and Drug Safety Research Group, Department of Pharmacy, School of Pharmacy, University of Oslo, Norway
[12]Division of Clinical Neuroscience, Department of Research and Innovation, Oslo University Hospital, Oslo, Norway
[13]Centre for Fertility and Health, Norwegian Institute of Public Health, Oslo, Norway
[14]Lead contact

#Correspondence: athina.samara@ki.se (A.S.), robert.lyle@medisin.uio.no (R.L.), ragnhild.eskeland@medisin.uio.no (R.E.)

[*] Current address: Department of Cancer Immunology, Institute for Cancer Research, Oslo University Hospital, and KG Jebsen Centre for B-cell malignancies, Institute for Clinical Medicine, University of Oslo, Norway.

[&] Current address: Department of Medical Genetics, Oslo University Hospital, and University of Oslo, Norway.

[§] Current address: Department of Analysis and Diagnostics, Section for Molecular Biology, Norwegian Veterinary Institute, Ås, Norway.

[+] Current address: Istituto di Genetica Molecolare, CNR - Consiglio Nazionale delle Ricerche, Pavia, Italy.

Supplemental Figures

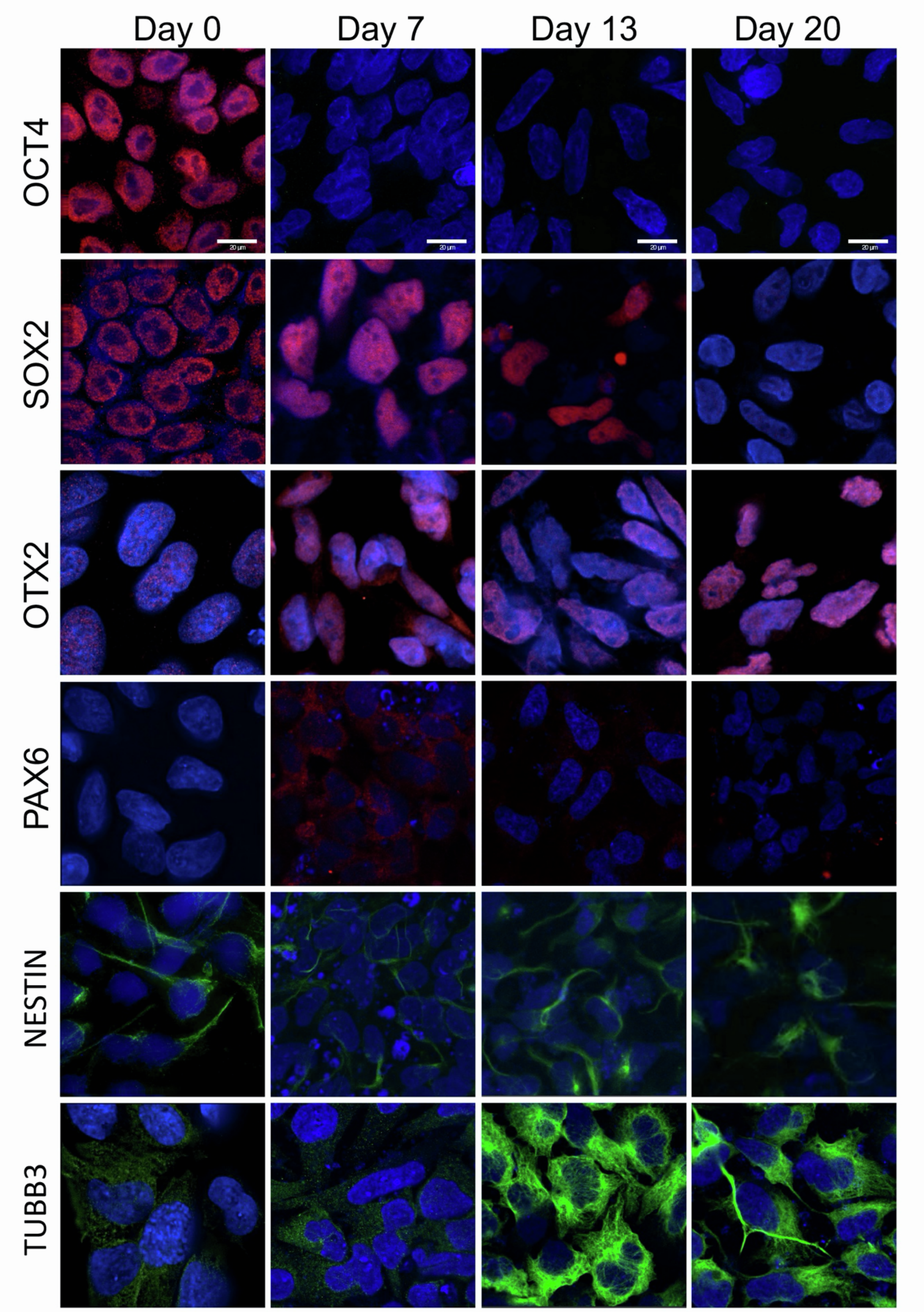

Figure S1. Immunofluorescence staining and imaging of pluripotency, neural and neuronal markers at Day 0, 7, 13 and 20 of differentiation, related to Figure 1. Confocal immunofluorescent micrographs show the localization of the transcription factors A) OCT4, B) SOX2, C) OTX2 and D)

PAX6, in red, and of the filamentous proteins E) NESTIN and F) β-III-TUBULIN, in green. The cells were counterstained with the DNA marker DAPI (blue), and the scale bar corresponds to 5 μm.

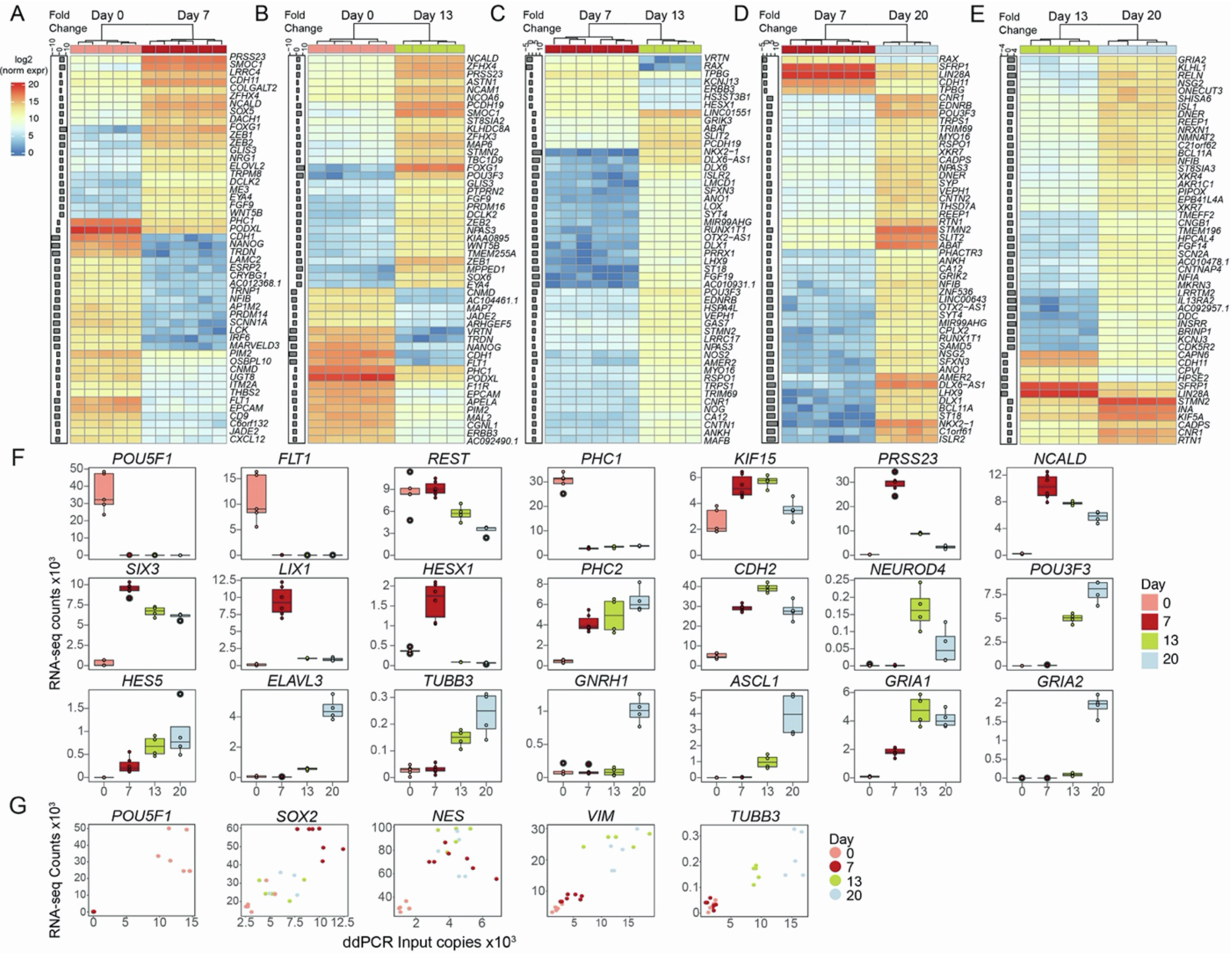

Figure S2. RNA-seq reveals novel marker genes during neuronal differentiation, related to Figure 2. A-E) Clustered heatmaps of the top 50 differentially expressed genes between timepoints A) Day 0 vs Day 7, B) Day 0 vs Day 13, C) Day 7 vs Day 13, D) Day 7 vs Day 20 and E) Day 13 vs Day 20. Fold change is shown to the left. F) Expression pattern of genes *POU5F1, FLT1, REST, PHC1, KIF15, PRSS23, NCALD, SIX3, LIX1, HESX1, PHC2, CDH2, NEUROD4, POU3F3, HES5, ELAVL3, TUBB3, GNRH1, ASCL1, GRIA1,* and *GRIA2* across differentiation. G) Correlation between read counts of the RNA-seq data and ddPCR input copies as shown in the scatter plots for marker genes *POU5F1, SOX2, NES, VIM* and *TUBB3*. Datapoints are color-coded in pink for Day 0, red for Day 7, green for Day 13 and blue for Day 20 in both G and H.

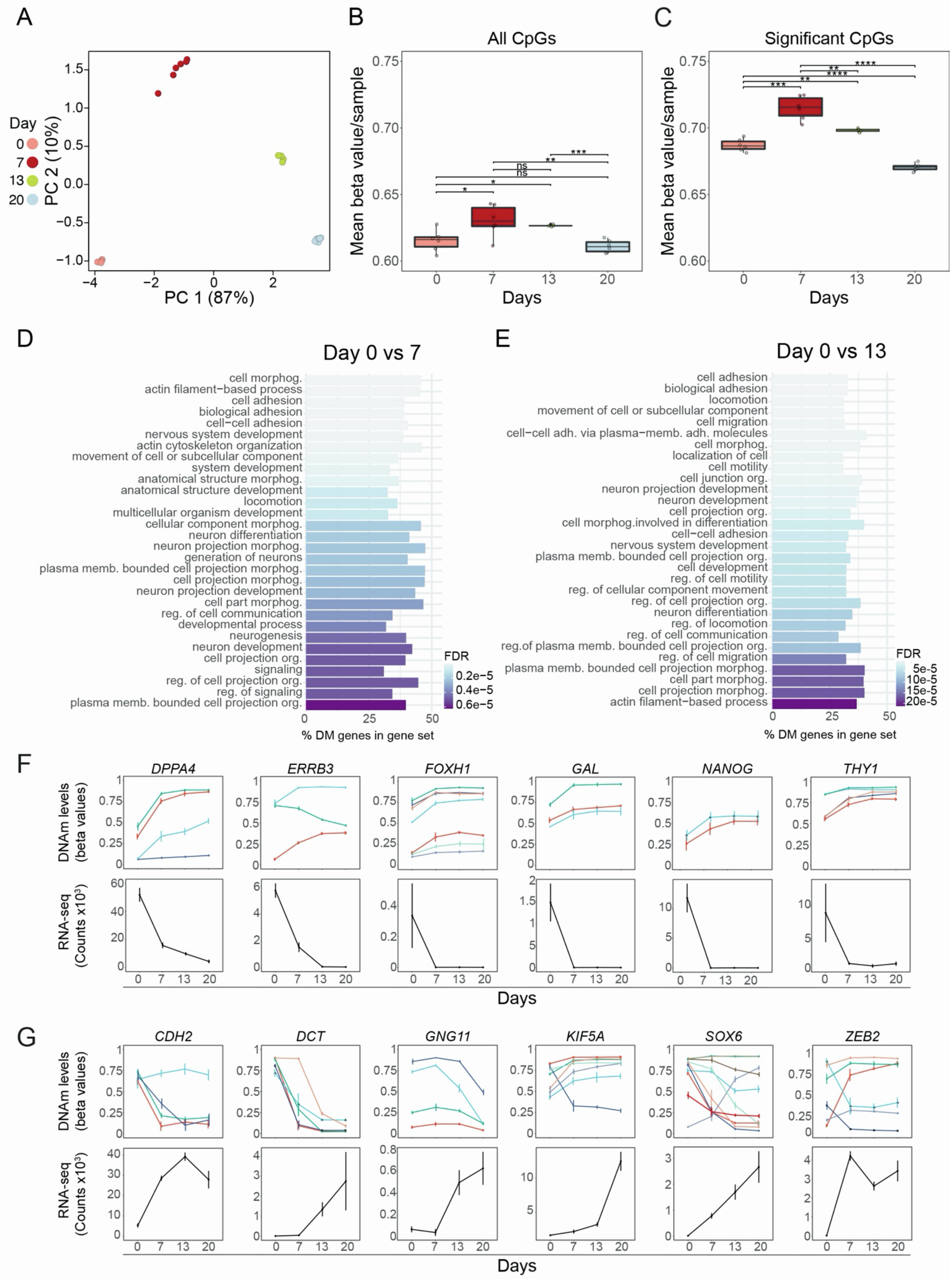

Figure S3. Genome-wide DNA methylation status changes during neuronal differentiation, related to Figure 3. A) Principal component analysis for all replicates for Days 0, 7, 13 and 20. B) Mean DNAm levels (beta-values) per sample for all CpGs across differentiation for Days 0, 7, 13 and 20. C) Mean DNAm levels (beta-values) per sample for all significant CpGs at Days 0, 7, 13 and 20. B-

C) *p < 0.05, **p < 0.01, ***p < 0.001, ****p < 0.0001. D-E) Top 30 ranked over-represented gene ontology (GO) terms in biological process (BP) (GO-BP) on the top 10% differentially methylated CpGs, regarding transition from Day 0 to Day 7 (D) and Day 0 to Day 13 (E). F) Examples of inverse correlation between DNAm and gene expression levels across neuronal differentiation, at the level of significant CpG regulators, as derived by the MORE analysis. For genes becoming repressed during differentiation, such as *DPPA4, ERRB3, FOXH1, GAL, NANOG* and *THY1,* DNAm levels increased as seen for some significant CpGs. Top panels show mean +/- standard deviation of DNAm and bottom panels show of normalized RNA-seq counts +/- standard deviation. G) MORE analysis of *CDH2, DCT, GNG11, KIF5A, SOX6* and *ZEB2,* with DNAm levels decreasing upon transcriptional activation during differentiation. Top panels show mean +/- standard deviation of DNAm and bottom panels show normalized RNA-seq counts +/- standard deviation.

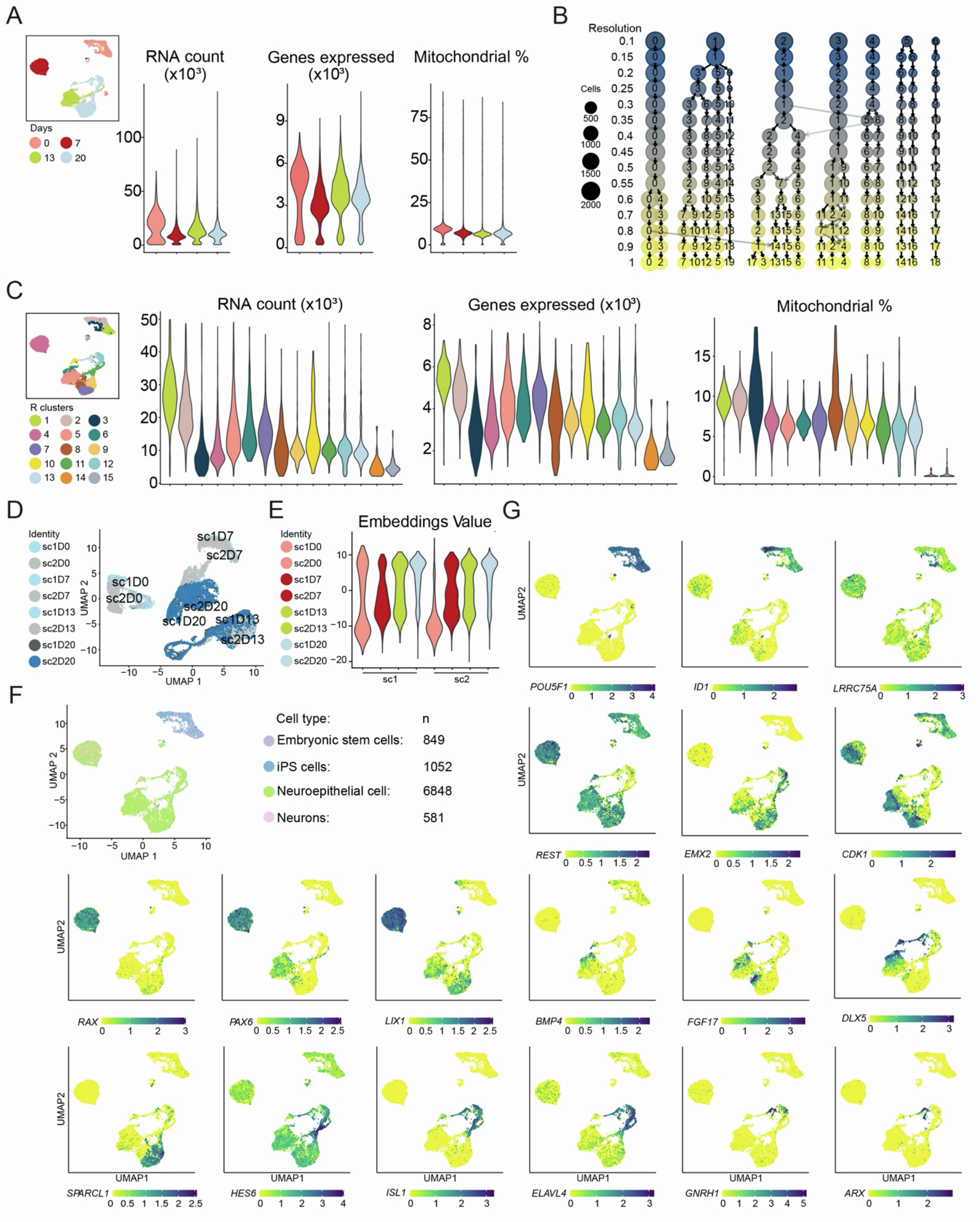

Figure S4. Quality control, clustering, and visualization of combined scRNA-seq datasets and genes used to profile the cell populations and annotate the clusters, related to Figure 4. A) UMAP showing original cell identity at day 0, 7, 13 and 20, and corresponding violin plots representing scRNA-seq count, number of genes expressed and mitochondrial content per cell before filtering. B) Dendrogram plot showing numbers of resulting clusters at resolutions 0.1 to 1, shown in colour gradient blue to yellow. Circle size represents the number of cells per cluster. C) UMAP showing clusters R1-15 at resolution 0.55 and the corresponding violin plots representing RNA count, number of genes expressed and mitochondrial content per cell after filtering. D) A Harmony UMAP on unfiltered

scRNA-seq replicates (coloured grey and blue). E) Harmony embedding plot on scRNA-seq replicates. F) UMAP projection of SingleR cell annotation to Human Primary Cell Atlas reference. The cell types listed are color coded and their frequency and numbers are included to the right. G). Representative UMAPs showing cluster specific or differentiation-driven gene expression across all four timepoints for *POU5F1, ID1, LRRC75A, REST, EMX2, CDK1, RAX, PAX6, LIX1, BMP4, FGF17, DLX5, SPARCL1, HES6, ISL1, ELAVL4, GNRH1* and *ARX*.

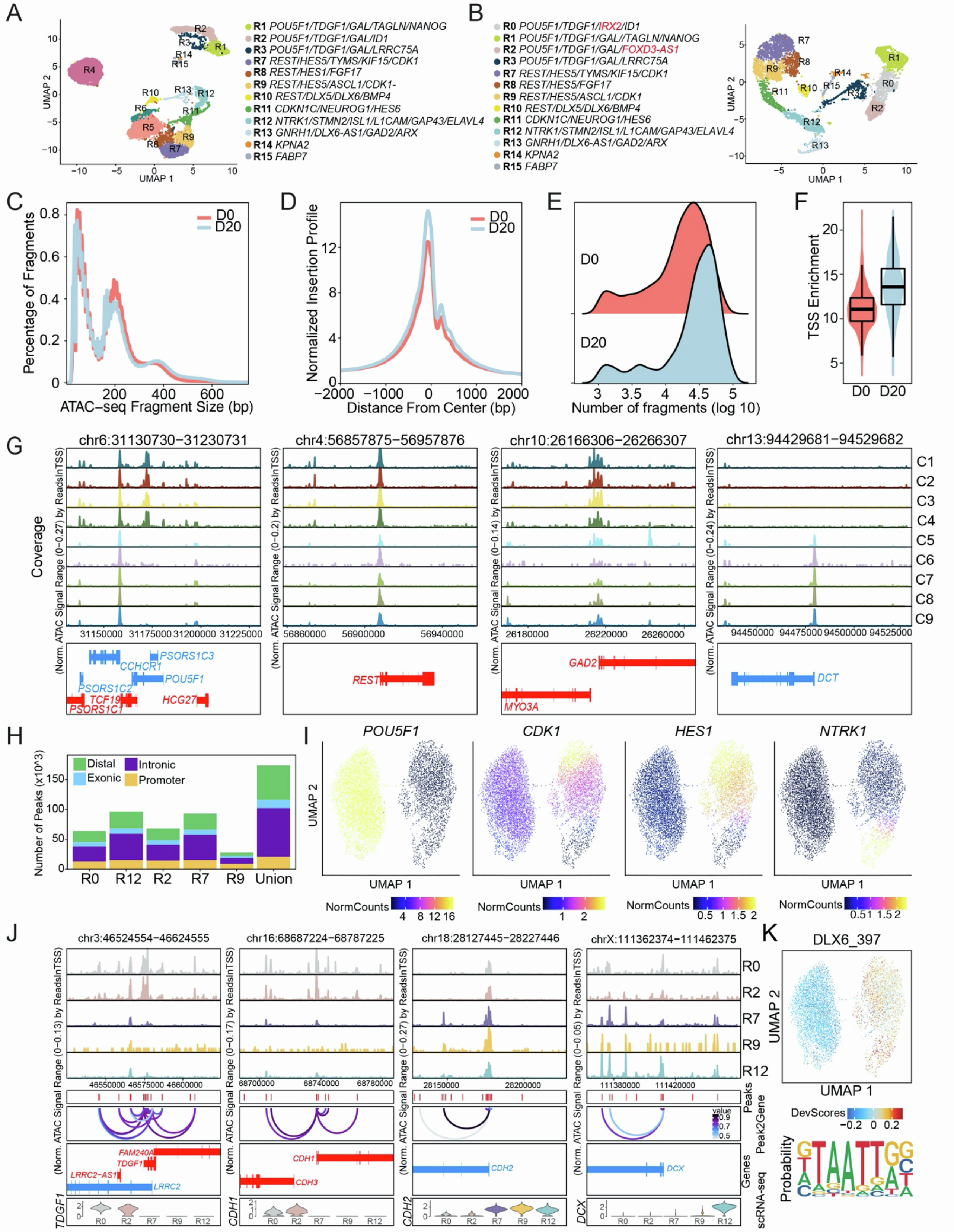

Figure S5. Gene expression and chromatin opening features during neuronal differentiation, related to Figure 5. A) Single-cell UMAP plot and cluster annotations for all timepoints Day 0, 7, 13 and 20. B) Single-cell UMAP plot and cluster annotations for timepoints Day 0 and 20. New cluster marker genes in red font. C) Percentage of ATAC-seq fragments and corresponding fragment size in base pairs for Day 0 and 20. D) Normalized insertion profiles and corresponding distance from centre in base pairs for Day 0 and 20. E) Number of fragments and F) TSS distribution of scATAC-seq datasets

represented as a box plot; where the middle represents the median, and the lower value is 25th percentile and the upper hinge is the 75th percentile of the data. Interquartile range (IQR) represent the distance between the upper and lower hinges and the whisker represents represent the lowest and largest values within 1.5 times the IQR. G) Genome track visualization of ATAC-seq per cluster for *POU5F1, REST, GAD2, CDH2* and *DCT* gene loci. H) Distribution of ATAC-seq peaks per cluster at promoter, intronic, exonic and distal regions for integrated clusters R0 (n=5027), R2 (n=448), R7 (n=2343), R9 (n=145), R12 (n=552), and all union peaks. I) UMAPs representing Gene Integration Matrix for *POU5F1, CDK1, HES1* and *NTRK1.* J) Genome tracks of ATAC-seq peaks in integrated clusters R0, R2, R7, R9 and R12 for *TDGF1, CDH1, CDH2* and *DCX* loci. Peaks and inferred peak-to-gene links for distal regulatory elements across the differentiation dataset are shown below. The bottom panel represents violin plots of expression of *TDGF1, CDH1, CDH2* and *DCX* in clusters R0, R2, R7, R9 and R12. K) Motif matrix UMAP for DLX6 with corresponding representative sequence logo.

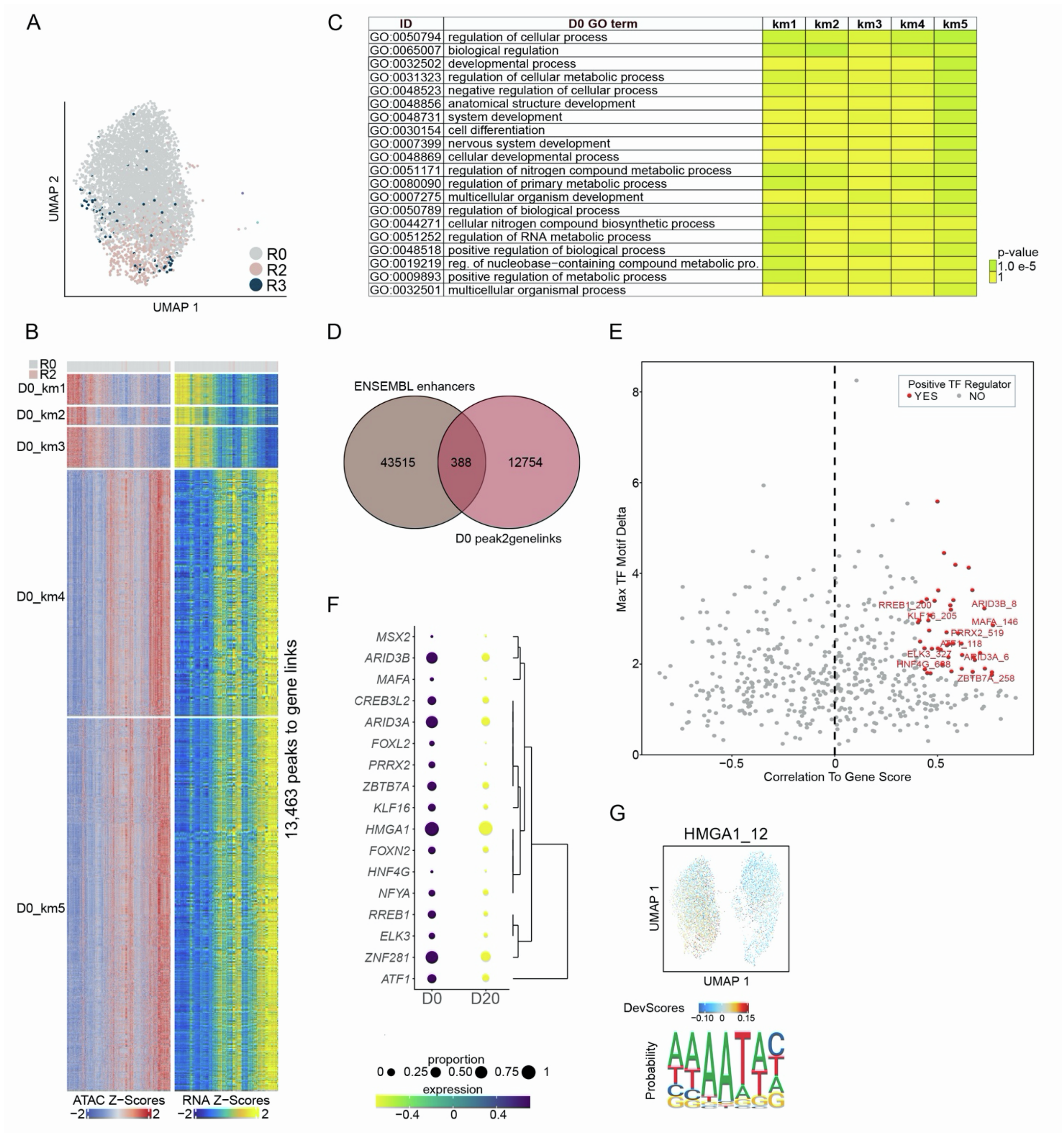

Figure S6. Molecular regulation of human embryonic stem cells. A) The constrained scATAC-seq Day 0 UMAP (Clusters R0, R2 and R3) B) A heatmap of chromatin accessibility and gene expression side-by-side representing 13, 463 peak-to-gene links in Day 0. Columns are annotated with colours above for integrated clusters R7, R9 and R12. Rows were clustered using k-means clustering (k = 5). C) Gene ontology analysis by GO profiler of Day 0 linked genes representing top GO terms for k- means 1-5. D) A venn diagram depicting correlation of Day 0 CREs with annotated enhancers in Ensembl Human Regulatory Regions (GRCh38.p13). E) A selection of gene integration value significant TFs with motifs in CREs are labelled with red circles. Not significant TFs have grey circles. F) A bubble plot representing expression of TF regulators in Days 0 and 20. G) Motif matrix UMAP and corresponding motif for *HMGA1*. See also Table S6.

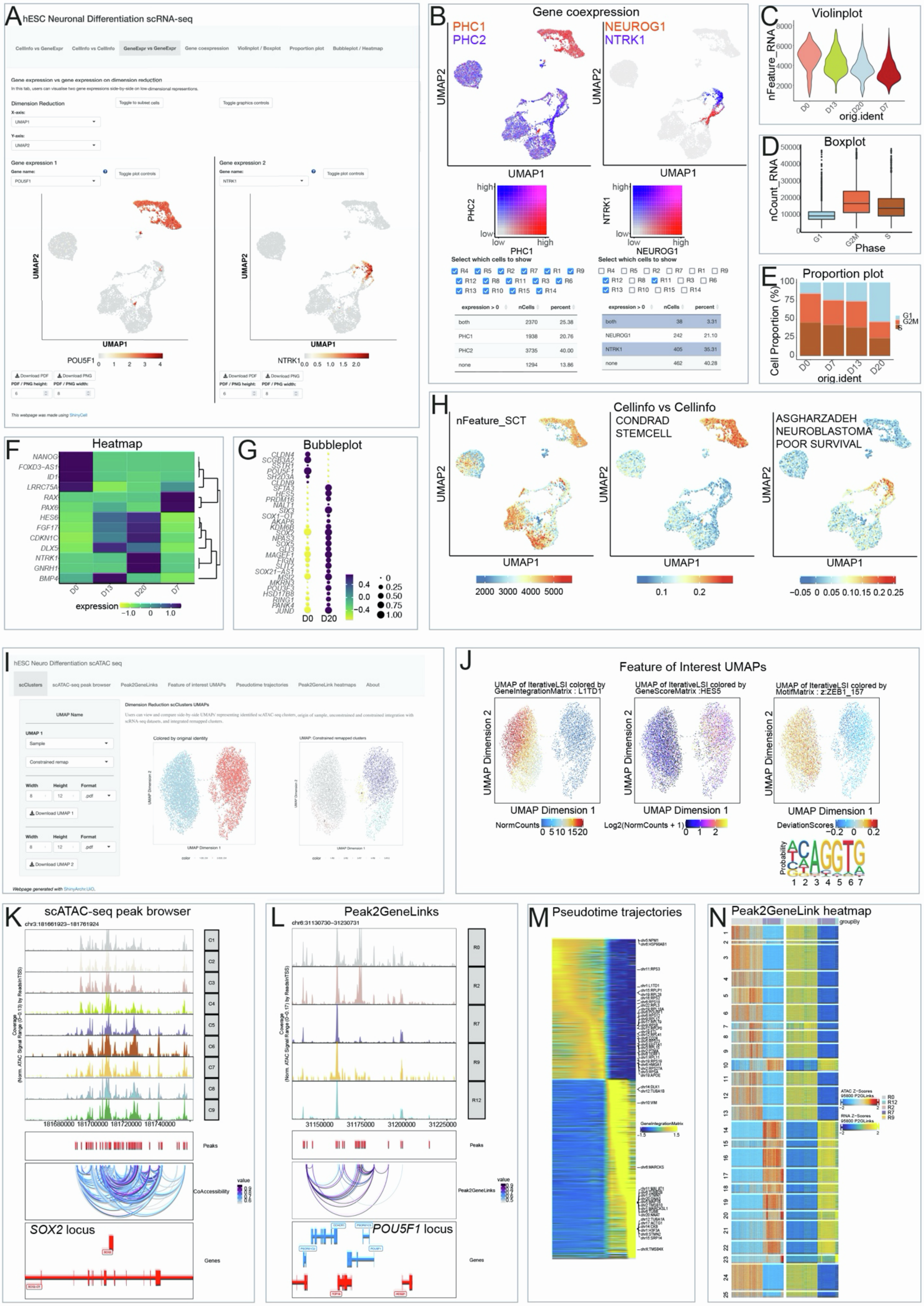

Figure S7. Visualizing gene expression and chromatin openness during neuronal differentiation at single-cell resolution in open access web interfaces, related to Figures 4 - 7 and S4-S6.

A) Visualization of the scRNA-seq data utilizing the ShinyCell tool (a screen dump). The user can explore the data in a web interface with seven different tabs. Gene expression can be explored for genes of interest in a tab together with cell information or in a gene expression tab as two side-by-side UMAPs, here shown for *POU5F1* and *NTRK1*. B) The gene co-expression tab allows inspection of overlapping expression of two genes in all clusters or selected clusters with downloadable number of cells and percentage data. Here illustrated for two gene pairs: *PHC1/PHC2*, and *NEUROG1/NTRK1* C) Violin plots, D) box plots and E) proportion plots can be generated to explore a number of features including cell cycle analysis and RNA count. The expression of a list of selected genes can be further explored by F) Heatmap or G) Bubble plot. H) In the cell info tab, the user can explore cellular features such as clusters, heterogeneity in the data by for example single-cell transcriptomics (SCT) and pathway analysis. We present another web interface that allows for exploration of chromatin openness utilizing our in-lab ShinyArchR.UiO tool. I) An illustration of the user interface of the ShinyArchR.UiO tool (a screen dump). The user can analyse the scATAC-seq data in six different tabs. In the scClusters tab, the user can visualize scATAC-seq data including original sample and cluster UMAPs. J) The feature of interest tab allows for exploration of gene score matrix, gene integration matrix and motif matrix UMAPs of selected genes, here shown for *L1TD1, HES5* and *ZEB1*. K) The user can also explore scATAC-seq clusters and integrated clusters in genome-browser views for selected gene loci with peaks and co-accessibility, here with an example from the *SOX2* locus. L) ATAC-seq peaks can also be explored across integrated clusters, as shown for the *POU5F1* locus. Finally, two tabs display different heatmaps including M) gene integration matrix as shown, but also gene score matrix, motif matrix and peak matrix can be explored side-by side. N) In the peak2genelink tab, a heatmap of gene scores from Days 0 and 20 scATAC-seq (to the left) with complementary gene expression from scRNA-seq data (to the right) are grouped and integrated clusters are represented by coloured of bars on top. Numbers 1-25 to the left of heatmap indicate groups/rows clustered by k-means. All plots can be downloaded as high-resolution PNGs or PDFs. A glossary for the two webtools are found in Table S7.

Supplemental Tables

Table S1. Global gene expression and DNA methylation changes between timepoints, related to Figs. 2, S2, 3, and S3*.

	Day 0 vs day 7	Day 0 vs day 13	Day 0 vs day 20	Day 7 vs day 13	Day 7 vs day 20	Day 13 vs day 20
# DE genes (down, up)	8972 (5025, 3946)	9252 (4948, 4304)	11313 (5710, 5603)	3602 (1624, 1978)	7120 (3258, 3861)	2379 (1529, 2084)
# DM CpGs (down, up)	161600 (24528, 137132)	146870 (46130, 100740)	210049 (94265, 115784)	39545 (31372, 8173)	122781 (105161, 17620)	47676 (45727, 1949)
# DM CpGs annotated to genes	110994	100816	143763	25337	79647	31082
# DMG overlapping with DEG (% of DEGs)	6446 (72)	6639 (72)	8388 (74)	1862 (52)	5117 (72)	1414 (59)
# DE genes regulated by CpGs (% of DEGs)	6441 (71)	6775 (73)	8011 (71)	2683 (74)	5230 (73)	1870 (79)
# Significant CpG regulators	16815	18950	21199	8072	14542	5651

*The number of differentially expressed genes (DEG) and differentially methylated (DM) CpGs are described for all comparisons. Of the DM CpGs that are annotated to genes, the overlap with DEG varies from 48 - 73%. The Multi-Omics regulations (MORE) analysis reveals the number of DEG that are regulated by CpGs and the number of CpG regulators.

Table S7. Glossary for the two webtools for easy access and exploration of the scRNA-seq and the chromatin accessibility data presented in this work. Related to Figs. 7 and S7.

**hESCNeuroDiff scRNA (https://cancell.medisin.uio.no/scrna/hescneurodiff/)**	
Orig.ident	Defined sample description.
nCountRNA	Number of RNA counts in each cell.
nFeatureRNA	Number of genes expressed in each cell.
Percent.mt	Mitochondrial percentage in each cell.
S.score	Cell cycle S phase analysis.
G2M score	Cell cycle G2/M phase analysis.
CC.difference	Regression score of cell cycle.
Phase	Cell cycle phase analysis.
Old.ident	Cluster number detected and named as default by Seurat with default threshold.
ncount.SCT	Normalized count after SCTransform.
nFeatureSCT	Normalized number of genes of SCTransform.
SCT_snn_res.0.1 …0.9	Cluster detection at resolutions 0.1 to 0.9.
Seurat_clusters	Cluster number detected and named as default by Seurat with the selected threshold.
Original_seurat_clusters	Seurat clusters default settings.
Pathway analysis	Pathway enriched terms.
PC1/2/3/4/5	Principal Component 1-5.
UMAP1/2	Uniform Manifold Approximation and Projection for dimension reduction 1 or 2.
tSNE1/2	t-distributed Stochastic Neighbour Embedding 1 or 2.
**hESCNeuroDiff scATAC (https://cancell.medisin.uio.no/scatac/hescneurodiff.archr/)**	
Orig.ident	Sample names.
Clusters	scATAC-seq clusters.
Unconstrained	Unconstrained scATAC-seq clusters.
Constrained	Integration of scATAC-seq clusters with original scRNA-seq clusters.
Constrained remap	Integration of scATAC-seq with annotated scRNA-seq clusters.
GeneScoreMatrix	scATAC-seq accessible peaks in vicinity of the gene based on custom ArchR distance-weighted accessibility models.
GeneIntegrationMatrix	Integration of scATAC-seq with scRNA-seq, where accessible peaks in vicinity of selected gene is linked with the measured gene expression.
PeakMatrix	Peak derived matrix of insertion counts of accessible regions.
MotifMatrix	TF motif enrichment within accessible peaks.
Co-accessibility	scATAC-seq correlations between two accessible peaks.
Peak2GeneLinkage	Integration of scRNA-seq and scATAC-seq showing correlations between peak accessibility and expressed genes.
Motif deviation score	Transcription Factor expression that is highly correlated with chromatin accessibility of the binding motif can be identified based on the correlation of the inferred gene score to the chromVAR motif deviation. Motif deviation score is computed per-cell deviations across all of motif annotations.
